# Patient-reported outcome measures poorly correlate with objective inflammatory bowel disease activity measures: a systematic review

**DOI:** 10.1093/ecco-jcc/jjaf132

**Published:** 2025-08-08

**Authors:** Xavier Calvet, Maria Giovanna Ferrario, Vanessa Marfil, Santos Armenteros, Manuel Barreiro-de Acosta

**Affiliations:** Gastroenterology Unit, Parc Taulí University Hospital, Parc Taulí Research and Innovation Institute (I3PT-CERCA), Sabadell, Spain; Department of Medicine, Autonomous University of Barcelona, Bellaterra, Spain; CIBERehd, Carlos III Health Institute, Madrid, Spain; Medical Statistics Consulting SL, Valencia, Spain; Medical Statistics Consulting SL, Valencia, Spain; Galapagos Biopharma Spain SL, Madrid, Spain; Inflammatory Bowel Disease Unit, University Clinical Hospital of Santiago de Compostela, Santiago de Compostela, Spain

**Keywords:** PROM, Crohn’s disease, ulcerative colitis

## Abstract

**Background and Aims:**

We investigated the correlations between patient-reported outcome measures (PROMs) and other measures of inflammatory bowel disease (IBD) activity.

**Methods:**

A systematic literature review was performed up to June 2022. Searches were conducted in PubMed, Scopus, and Web of Science. A descriptive analysis was performed. The search protocol was registered in PROSPERO (CRD42022383899).

**Results:**

Nineteen studies assessed correlations between PROMs and clinical, endoscopic, and laboratory measures of disease activity in IBD. In Crohn’s disease (CD), weak positive correlations were reported for PROMs (eg, the 2 item patient-reported outcome [PRO-2], mobile Health Index [mHI] for CD) and endoscopic scores, more often the Simple Endoscopic Score for CD (SES-CD). In ulcerative colitis (UC), PROMs like PRO-2, the Monitor IBD at Home rectal bleeding item, and the mHI showed weak-to-moderate correlations with the Mayo endoscopic subscore (MES). PROMs also demonstrated limited concordance with laboratory measures such as fecal calprotectin (FCP) and C-reactive protein (CRP) in both CD and UC. The substantial heterogeneity in study designs precluded a structured analysis.

**Conclusions:**

Although current PROMs offer valuable complementary insights into IBD control from the patient’s perspective, they cannot replace objective measures of IBD activity. Future research should focus on refining PROMs and generating composite indices to improve their accuracy and usefulness.

## Graphical abstract

**Graphical Abstract:**
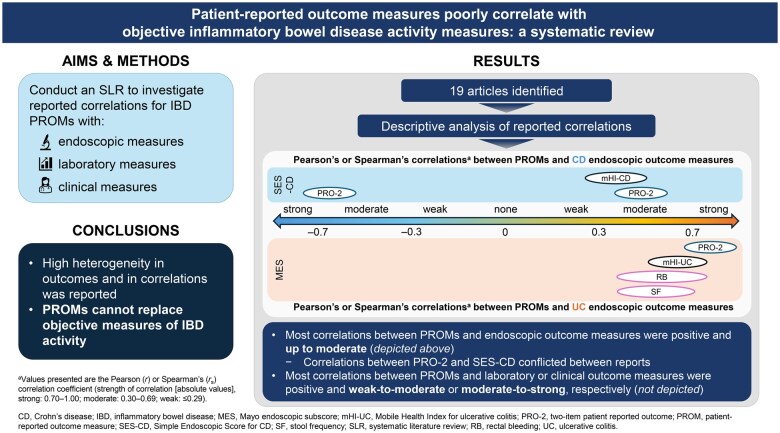

## 1. Introduction

Inflammatory bowel disease (IBD) is characterized by flares of bowel inflammation leading to heterogeneous symptoms that follow a chronic and recurrent course.^1^ The term IBD includes 2 main diseases: Crohn’s Disease (CD) and ulcerative colitis (UC).^2^^,^^3^

In IBD management, patient-centered care is increasingly important. Healthcare professionals are no longer requested to direct patient care; by contrast, they should recognize patients’ autonomy by providing accurate information allowing patients to make their own therapeutic decisions tailored to individual needs and preferences.^4^^,^^5^ This approach improves clinical results^6–8^ and increases patient satisfaction and treatment adherence.^9–11^

Patient-centered care includes measuring IBD activity from the patient’s point of view using patient-reported outcomes (PROs). PROs are defined as any outcome directly reported by the patient without the interpretation of a clinician. They capture information about the patient’s health, quality of life, or functional status associated with healthcare or treatment.^12^ Specific, validated and standardized, self-completed instruments to assess PROs are termed patient-reported outcome measures (PROMs). PROMs are intended to support clinical decisions, to complement other measurements of disease activity, to compare treatment outcomes, and to evaluate medical policies and practices.^13^ PROMs are increasingly used in IBD to help to monitor disease, in both clinical trials and clinical practice.^14^^,^^15^ They are also used to identify which outcomes matter most to each patient, which is crucial in providing patient-centered care.^16^ Nonetheless, there is some controversy regarding the use of PROMs in the management of IBD and their clinical validity. The main concerns are subjectivity and variability of self-reported data and the potentially limited sensitivity of PROMs in the detection of subtle changes in disease activity.^17^

In fact, imaging techniques, especially endoscopy, are accepted as the most reliable and objective tools to evaluate IBD activity because they allow direct evaluation of mucosal inflammation. In IBD, endoscopy holds a key position in diagnosing the severity and extent of mucosal lesions and/or assessing mucosal healing and, therefore, guiding therapeutic decisions. Simple Endoscopic Score for Crohn’s Disease (SES-CD)^18^ and the Crohn’s Disease Endoscopic Index of Severity (CDEIS)^19^ are common endoscopic indices used in CD, while Mayo endoscopic subscore (MES) is the index most frequently used in UC.^20^ Endoscopy, however, is an invasive and expensive technique. Therefore, patient discomfort and risks related to endoscopy prevent its use for regular routine follow-up. For this reason, clinicians rely on a series of indirect measurements of disease activity.^21^ These measurements include inflammatory biomarkers (eg, C-reactive protein [CRP] and fecal calprotectin [FCP]) and activity indices assessed by the clinician (eg, the Harvey-Bradshaw Index [HBI],^22^ the CD Activity Index [CDAI],^23^ and the Simple Clinical Colitis Activity Index [SCCAI]^24^). In conclusion, while endoscopic indices allow direct evaluation of mucosal inflammation and are accepted as the gold standard for evaluating IBD activity, their limitations require noninvasive tools to monitor CD disease activity. Between these tools, PROMs are gaining popularity, although their usefulness to predict endoscopic activity is controversial.

The aim of this study was to assess the reliability of IBD-specific PROMs in measuring disease activity by analyzing the correlation and/or association of these PROMs with validated clinical, laboratory, or endoscopic IBD activity measures.

## 2. Materials and methods

### 2.1 Literature search strategy

A systematic literature review was carried out to identify relevant studies focusing on the development, validation, or utilization of PROMs designed to monitor clinical manifestations in adult patients with IBD. The search was performed in PubMed, Scopus, and the databases included in the Web of Science, up to June 30, 2022. Also, multiple searches were performed in Google Scholar to confirm the searches were exhaustive. The search strategy utilized a Boolean combination of terms as detailed in Supplementary Table 1. Specific search strategies tailored to each database are provided in Supplementary Table 2.

### 2.2 Study selection

Following the initial search, articles were screened for eligibility according to predefined criteria. Studies were excluded if (1) the PROM analyzed was not validated in the IBD population, (2) if the PROM was not available in English or Spanish, (3) if the PROM was not used to measure study outcomes (ie, used solely for baseline patient classification), (4) if they were not original research articles, (5) if the PROM measured aspects unrelated to disease activity (eg, quality of life, or social or work performance), (6) if the PROM was used for pediatric populations, (7) if the PROM was designed for a specific condition that is not applicable for most patients with IBD (eg, PROM designed for ileorectal pouch evaluation or fecal incontinence), or (8) if the PROM was a generic instrument not specifically targeting IBD patients.

The screening, selection, and inclusion processes were carried out by 3 investigators, with a fourth investigator overseeing the selection process. Any disagreements or uncertainties regarding inclusion were discussed and resolved through consensus, or by consulting the fourth reviewer when necessary.

### 2.3 Data extraction

Data extraction was performed by the same 3 investigators, using a standardized extraction table in Excel format. The extracted data included details on study characteristics, participant demographics, PROM characteristics, and outcomes related to PROM validation and utilization.

### 2.4 Descriptive analysis of correlation and predictive performance

The reported correlations between PROMs and other IBD activity measures were identified from each selected article and reported. Correlations were classified based on the absolute Pearson or Spearman’s correlation coefficients as follows: strong (0.70-1.00), moderate (0.30-0.69), and weak (below 0.30).

Also, predictive performance indicators were extracted and reported. Measures included: sensitivity, specificity, positive predictive value (PPV), and negative predictive value (NPV). In some articles, the area under the receiver operating characteristic curve (AUROC) was used for comparing each PROM with other measures of disease activity, such as the proportion of patients in endoscopic remission. An AUROC of 0.50 indicated no discrimination, 0.60-0.70 was considered acceptable, 0.80-0.90 was considered good, and 0.90 or greater was excellent. Additionally, inter-observer agreement was reported in some studies using Cohen’s kappa. The interpretation of kappa values was as follows: less than 0 (no agreement beyond chance), 0-0.20 (slight agreement), 0.21-0.40 (fair agreement), 0.41-0.60 (moderate agreement), 0.61-0.80 (substantial agreement), and 0.81-1.00 (almost perfect agreement).

### 2.5 Quality assessment

The critical appraisal skills programme (CASP) tool was employed to assess the quality of evidence presented in each included study.^25^ This tool facilitated the evaluation of study design, methodology, and potential biases.

### 2.6 Protocol registration and reporting guidelines

The search protocol was registered in PROSPERO (CRD42022383899).^26^ The systematic literature review adhered to the Preferred Reporting Items for Systematic Reviews and Meta-Analyses (PRISMA) guidelines to ensure transparency and completeness in reporting the review process.^27^

## 3. Results

### 3.1 Selection process and study population description

**Figure 1.**
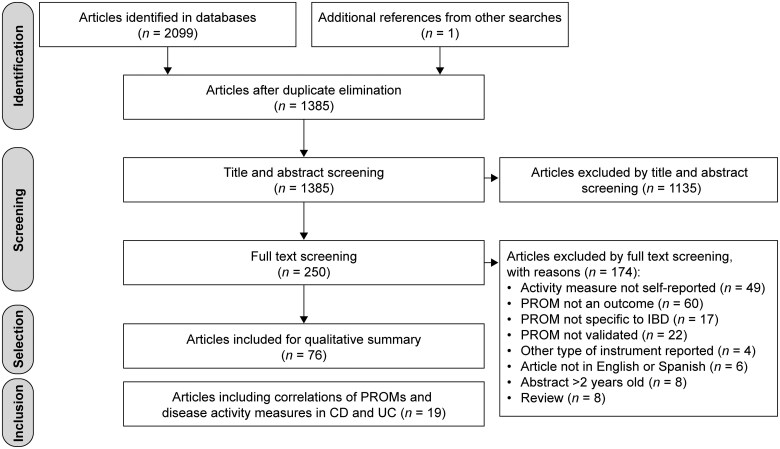
PRISMA flowchart: results from systematic search and article selection. CD, Crohn’s disease; IBD, inflammatory bowel disease; PROM, patient-reported outcome measure; UC, ulcerative colitis.

A summary of the systematic search strategy and article selection is shown in Figure 1. Initially, 2099 articles were found by database and manual searches, while an additional article, not previously identified, was manually added when performing free searches in Google Scholar. After elimination of duplicates, 1385 unique articles were identified. Subsequent screening of titles, abstracts and full texts led to the selection of 76 articles that reported the use of PROMs for measuring IBD activity. Reasons for article exclusion are reported in Supplementary Table 3. Of the 76 articles selected, 22 articles were independently identified by 3 reviewers in parallel. Three of these 22 articles were subsequently excluded following assessment by the fourth reviewer, because they evaluated the long-term prognostic value of PROM without a direct comparison with endoscopic, clinical, or biological activity. The remaining 19 articles fulfilled inclusion criteria because they analyzed correlations between PROMs and other disease activity measures, and/or the accuracy of PROMs in predicting remission or healing measures and were thus included in the final selection.^28–46^

### 3.2 Characteristics of included articles

Supplementary Table 4 summarizes the design features of the 19 included articles and relevant validation studies, including those tools that were not validated within the same study.^28–46^ Among these, 4 studies included a CD population only, 5 included a UC population only, while the remaining 10 studies included both CD and UC patients. Fifteen studies had a real-world design (10 prospective cohort studies; 5 cross-sectional studies) and 4 were *post hoc* analyses of randomized controlled trials or real-world studies.

Evaluation of study quality using the CASP checklist revealed low-to-moderate quality across most studies, as depicted in Supplementary Table 5.

### 3.3 Characteristics of included PROMs

Among the 19 included articles, 16 different validated IBD-specific PROMs were identified (Supplementary Table 6). Seven of these were utilized across IBD populations, whereas 3 and 6 were specific for CD and UC, respectively. The PROMs included the assessment of various signs and symptoms of IBD activity, of which the most reported signs and symptoms were stool frequency (SF; day and night), abdominal pain (AP) or discomfort, frequency of loose stools/diarrhea, rectal bleeding (RB), urgency of defecation, general well-being, and fatigue. Additional aspects assessed were anal fissures, active fistulas and/or abscesses, and extracolonic manifestations, including arthralgia, red or swollen joints, uveitis, erythema nodosum, pyoderma gangrenosum, and aphthous ulcers. Additionally, certain PROMs also included aspects of health-related quality of life, such as the impact on emotional components and daily activities.

### 3.4 Concordance between PROMs and endoscopic measures of disease activity

For CD, 6 studies reported data on the correlation between PROMs and endoscopic activity measures and/or the diagnostic accuracy of PROMs for detecting disease activity (Table 1).^28–33^ A single study published by Dragasevic et al. reported a strong inverse correlation between the PRO-2 and the SES-CD in 63 patients with CD and colonic involvement.^29^ Most of the remaining studies described a weak-to-moderate positive correlation between PROMs and endoscopic scores, ranging from 0.29^32^ to 0.36.^30^ Sensitivity to detect endoscopic activity ranged from 23%^29^ to 68%^28^; specificity ranged from 81%^28^ to 98%.^29^ Finally, AUROCs ranged from 0.54 for the HBI to 0.70 for the patient-reported opinion on whether IBD is active.^33^ In addition, Zittan et al. demonstrated that the combination of HBI with other outcome measures was not a useful surrogate to detect endoscopic remission.^33^

**Table 1.** Descriptive analysis of articles evaluating the correlation and/or association/predictive accuracy of PROMs with endoscopic scores in IBD

CD					
Article	Number of patients (*N*)	PROM	Correlation/association/predictive accuracy measures	Correlation with SES-CD^a^	Association and predictive accuracy with respect to SES-CD
de Jong MJ et al. 2019^28^	135	MIAH^c^	Validation phase: MIAH-CD questionnaires’ sensitivity, specificity, PPV, and NPV	NA	Sensitivity = 67.6% Specificity = 81.3% PPV = 69.3% (with cut-off at 3.6) NPV = 80.0% (with cut-off at 3.6)
Dragasevic S et al. 2020^29^	63	PRO-2^b^	Spearman’s correlation coefficients. Sensitivity and specificity were also measured against endoscopy	*r_s_* = −0.67, *p *< 0.05	Sensitivity = 22.5% (95% CI: 10.8-38.4%) Specificity = 97.7% (95% CI: 71.9-98.9%) PPV = 81.8% (95% CI: 51.5-95.0%) NPV = 40.4% (95% CI: 35.5-45.5%)
Lewis JD et al. 2020^30^	353	PRO-2^b^	Pearson correlation coefficients	PRO-2: *r* = 0.36 (95% CI: 0.27-0.45), *p* < 0.001	NA
Morris MW et al. 2018^31^	164	PRO-2 PRO-3	AUROC curves to evaluate active disease using SES-CD at 2 levels: (SES-CD ≥7 and SES-CD ≥2 for moderate disease). Sensitivity, specificity	NA	PRO-3: SES-CD ≥7; AUROC = 0.56 (95% CI: 0.55-0.57); sensitivity = 63%; specificity = 88% PRO-2: SES-CD ≥7; AUROC = 0.56 (95% CI: 0.55-0.57); sensitivity = 61%; specificity = 55% PRO3: SES-CD ≥2; AUROC = 0.66 (95% CI: 0.65-0.67); sensitivity = 34%; specificity = 94% PRO-2: SES-CD ≥2; AUROC = 0.58 (95% CI: 0.57-0.59); sensitivity = 38%; specificity = 85%
Van Deen WK et al. 2016^32^	301	mHI-CD^d^	Spearman’s correlation coefficient, ROC curves	*r_s_* = 0.29, *p *< 0.039	AUROC: 0.632 (CI not provided)
Zittan E et al. 2017^33^	88	HBI-PRO PROp	ROC curves	NA	PROp: AUROC of 0.70 for predicting SES-CD remission HBI had an AUROC = 0.54 for predicting SES-CD endoscopic remission

UC					
Article	Number of patients (*N*)	PROM	Correlation/association/predictive accuracy measures	Correlation with MES^a^	Association and predictive accuracy for remission (MES = 0 or 1)
Colombel JF et al. 2017^38^	103	PRO-2	PPV, NPV, sensitivity, and specificity of RB and SF subscores for endoscopic remission (MES = 0/1) were calculated for each definition separately for RB subscore = 0, SF subscore = 0, and RB + SF subscore = 0	NA	RB = 0: PPV = 60.0% (95% CI: 45.9-73.0) NPV = 89.6% (95% CI: 77.3-96.5) Sensitivity = 86.8% (95% CI: 71.9-95.6) Specificity = 66.2% (95% CI: 53.4-77.4) SF = 0: PPV = 72.5% (95% CI: 56.1-85.4) NPV = 85.7% (95% CI: 4.6-93.3) Sensitivity = 76.3% (95% CI: 59.8-88.6) Specificity = 83.1% (95% CI: 71.7-91.2) RB + SF = 0 PPV = 74.3% (95% CI: 56.7-87.5) NPV = 82.4% (95% CI: 71.2-90.5) Sensitivity = 68.4% (95% CI: 51.3-82.5) Specificity = 86.2% (95% CI: 5.3-93.5)
de Jong MJ et al. 2019^28^	Development: 80, Validation: 131	SF and RB (MIAH^c^)	Spearman correlation coefficients were calculated between individual items and endoscopic disease activity in the development phase. Sensitivity, specificity, PPV, and NPV for MIAH-UC were calculated in the validation phase, defining remission as MES 0/1	*r_s_* (RB) = 0.58, *p *< 0.001 *r*_s_ (SF) = 0.51, *p *< 0.001	Sensitivity = 68.2% Specificity = 80.5% PPV = 63.8% (with cut-off at 3.5) NPV = 83.3% (with cut-off at 3.5)
Dragasevic S et al. 2020^29^	96	PRO-2^b^	Spearman’s correlation coefficient. Sensitivity, specificity, PPV, and NPV for endoscopic remission	*r_s_* = 0.84, *p *< 0.05	Specificity = 99% Sensitivity = 45% PPV = 96% NPV = 59%
Golovics PA et al. 2021^39^	171	PRO-2	AUROC, sensitivity, specificity, PPV, NPV to assess remission (MSC = 0/1)	NA	AUROC: PRO-2 = 0.868 SF = 0.842 RB = 0.814 pMCS = 0.927 PRO SF = 0 versus MES ≤1 Sensitivity = 89.0% Specificity = 74.4% PPV = 91.1% NPV = 69.6% PRO RB = 0 versus MES = 0 Sensitivity = 97.9% Specificity = 45.2% PPV = 70.4% NPV = 94.3% PRO RB = 0 versus MES ≤1 Sensitivity = 95.3% Specificity = 67.4% PPV = 89.6% NPV = 82.9% PRO-2 remission Sensitivity = 92.1% Specificity = 76.7% PPV = 92.1% NPV = 76.7% pMCS Sensitivity = 97.6% Specificity = 74.4% PPV = 91.9% NPV = 91.4%
Ma C et al. 2020^41^	817	PRO-2^b^	Spearman’s rank correlation coefficient with 95% CI. Sensitivity, specificity, PPV, and NPV of RB and SF for endoscopic remission (MES = 0/1) at week 8 and week 38 separately for PROs with RB = 0, SF = 0, and RB + SF = 0 as criteria	*r_s_* (RB) = 0.39 (95% CI: 0.32-0.45), *p *< 0.001 *r_s_* (SF = 0.34 (95% CI: 0.27-0.40), *p* < 0.001	Remission (MES = 0 or 1): RB+SF (week 38): Sensitivity = 59.5% Specificity = 82.2% PPV = 72.7% NPV = 71.7%
Van Deen WK et al. 2016^32^	265	mHI-UC^d^	Spearman’s correlation coefficient, AUROC, sensitivity, specificity, PPV, and NPV to predict endoscopic remission (MES = 0/1). ROC analysis	*r_s_* = 0.60, *p *< 0.0001	AUC = 0.82 (with a 3.2 cut-off) Sensitivity = 85% (with a 3.2 cut-off) Specificity = 80% (with a 3.2 cut-off) NPV = 95% PPV = 55% (with a 3.2 cut-off)

a The correlations provided were performed using an MES of 0 or 1 as the definition for remission. Scales vary across the studies that use different coefficients and should not be compared directly. Correlations were considered strong when the absolute Pearson (*r*) or Spearman’s (*r*_s_) correlation coefficient lay between 0.70 and 1.00, moderate when the coefficient lay between 0.30 and 0.69, and weak when the coefficient was less than or equal to 0.29.

b PRO-2 was based on RB and SF for UC and abdominal pain and number of liquid or very loose stools and abdominal pain for CD.

c The MIAH questionnaire assessed IBD-specific items of abdominal pain, fatigue, fever, frequency of liquid or soft stools, general well-being, mucus loss, patient-reported disease activity, perceived stress, RB, SF (day), SF (night), urgency of defecation, and weight loss.

d The mHI-CD and mHI-UC assessed abdominal pain, anorexia, disease activity, fever, general well-being, nausea/vomiting, SF, stool consistency, RB, and urgency.

Abbreviations: CD, Crohn’s disease; CI, confidence interval; IBD, inflammatory bowel disease; MES, Mayo endoscopic subscore; mHI-CD, mobile Health Index for CD; mHI-UC, mobile Health Index for UC; MIAH, Monitor IBD at Home; pMCS, partial Mayo Clinic Score; PRO-2, 2 item patient-reported outcome; PROp, patient-reported opinion on whether CD is active; PROM, patient-reported outcome measure; *r*, Pearson correlation coefficient; RB, rectal bleeding; *r*_s_, Spearman’s correlation coefficient; SES-CD, Simple Endoscopic Score for CD; SF, stool frequency; UC, ulcerative colitis.

For UC, 6 studies reported data on the correlation between a PROM and endoscopic activity measures, and/or the diagnostic accuracy of PROMs for detecting endoscopic activity.^28^^,^^29^^,^^32^^,^^38^^,^^39^^,^^41^ Correlations between PROMs and the MES were positive and moderate to strong, ranging from 0.34^41^ to 0.84.^29^ PROMs generally showed a moderate-to-high sensitivity and specificity to detect endoscopic activity: sensitivity ranged from 45%^29^ to 98%^39^ and specificity from 45%^39^ to 99%.^29^ Finally, AUROCs ranged from 0.82^32^ to 0.93.^39^ Regarding individual items, RB subscores generally demonstrated a noticeably higher accuracy when compared to SF.^38^^,^^39^^,^^41^

### 3.5 Concordance between PROMs and laboratory measures of disease activity

Six studies evaluated the concordance between PROMs and different laboratory measures,^35^^,^^36^^,^^39^^,^^40^^,^^43^^,^^44^ mainly CRP and FCP (Table 2). Concordance was weak-to-moderate for PROMs and biological markers of activity. In CD, neither the IBD-10 nor the HBI effectively distinguished patients with increased FCP levels (>250 µg/g) from those with normal levels.^43^ The Numerical Rating Scale (NRS) did not correlate with CRP, erythrocyte sedimentation rate (ESR), or FCP (Table 2).^44^ ­Similar limitations were observed in UC patients. Neither the IBD-10 nor the SCCAI satisfactorily discriminated patients with normal vs. elevated FCP levels.^43^ CRP showed fair inter-rater agreement with the Patient Simple Clinical Colitis Activity Index (P-SCCAI) and the SCCAI, while a substantial agreement was found between the Physician Global Assessment (PGA) and both P-SCCAI and SCCAI.^35^ FCP correlated moderately with the 8-item questionnaire, IBD-Control-8, and the VAS, IBD-Control-VAS (Table 2).^36^

**Table 2.** Concordance between PROMs and clinical and/or laboratory measures of disease activity.

CD						
Article	Number of patients (*N*)	PROM	Disease activity measure	Correlation/association/predictive accuracy measures	Correlation	Association and predictive accuracy
Bennebroek Evertsz KA et al. 2013^34^	181	P-HBI	HBI	Agreement between HBI and P-HBI was calculated with Spearman’s *r*. Agreement regarding active disease versus remission was calculated by percent agreement and Cohen’s κ. Sensitivity, specificity, PPV, and NPV were calculated from the article data	*r_s_* = 0.82	Cohen’s κ = 0.52 Sensitivity = 74% Specificity = 91% PPV = 96% NPV = 53%
Bodger K et al. 2013^36^	160	IBD-­Control	HBI Physician global assessment (PGA). Composite evaluation for “quiescent disease”	Correlation was expressed as Spearman’s *r_s_* values. A negative correlation was expected (the higher the IBD-c, the lower the symptoms score). AUROC, sensitivity, specificity, PPV, and NPV to predict “quiescent diseases” pooled for UC and CD	IBD-Control-8: vs. HBI: *r_s _*= −0.68, *p* < 0.01 vs. PGA: *r*_s _= −0.45; *p* < 0.01 IBD-Control-VAS: vs. HBI: *r_s _*= −0.60, *p* < 0.01 vs. PGA: *r_s _*= −0.47, *p* < 0.01	NA
Clara I et al. 2009^37^	184	MIBDI	HBI	Sensitivity and specificity for detecting activity (HBI ≥4) were provided at 3 time periods (0, 12, and 24 months). PPV and NPV were calculated using the data provided in the study	NA	At 0, 12, and 24 months, respectively: Sensitivity = 88%, 85%, and 84% Specificity = 51%, 57%, and 66% PPV = 78%, 75%, and 77% NPV = 67%, 72%, and 75%
Sexton KA et al. 2019^42^	124	IBDSI (long form, LF) Self-reported HBI	Nurse/clinician-­administered HBI, PGA	Pearson correlation analyses, AUROC. Sensitivity, specificity, PPV, and NPV using HBI ≥5 or PGA	IBDSI (LF) vs. PI: *r = *0.86 IBDSI (LF) vs. PGA: *r* = 0.70 Self-reported HBI vs. nurse/clinician administered HBI: *r *= 0.86	IBDSI (LF) vs. HBI AUROC = 0.93 Sensitivity = 84% Specificity = 89%
Subramanian S et al. 2016^43^	142	IBD-10 NRS	HBI CRP FCP	Spearman’s rank correlation coefficients. AUROC. Sensitivity, specificity for HBI-defined remission (≤4) Mann-Whitney U test for comparing values of IBD-10 between patients with CRP >5 vs. normal and FC >250 vs. normal	IBD-10 vs. HBI: *r_s _*= −0.69, *p* < 0.001 IBD-10 scores were similar in patients with normal vs. elevated FC and normal vs. elevated CPR	AUROC = 0.86 (95% CI: 0.81-0.91; *p *= 0.035) Sensitivity = 95% (95% CI: 86-94) Specificity = 75% (95% CI: 67-82)
Surti B et al. 2013^44^	Study A: 46 Study B: 135	IBD NRS	CDAI HBI CRP ESR FCP	Pearson’s correlation coefficient	Study A: vs. CDAI: *r* = 0.59, *p* < 0.0001 vs. HBI: *r* = 0.32, *p* < 0.0001 Study B: vs. HBI: *r* = 0.25, *p* < 0.0001 vs. CRP no significant correlation	NA
Tow KE et al. 2019^45^	48	PRO-2, PRO-3	MHBI	Spearman’s rho	PRO-2: *r* = 0.95, *p* < 0.001 PRO-3-CD: *r* = 0.88, *p* <0.001 PRO-2-CDSR: *r * = 0.81, *p* < 0.001 PRO-3-CDSR: *r * = 0.79, *p* < 0.001	NA
Van Deen WK et al. 2016^32^	110	mHI-CD	HBI	Spearman’s correlation coefficient, AUROC analysis for detecting active disease (HBI >4)	*r_s_* = 0.75, *p* < 0.0001	AUROC = 0.90 Sensitivity = 94% Specificity = 67%
Vicente Lidón et al. 2021^46^	177	IBD-Control	HBI Composite evaluation for “quiescent disease”	Spearman’s correlation coefficients. AUROC. Sensitivity and specificity for “quiescent disease” (data pooled, separate data for CD and UC were not provided)	Moderate negative correlation with the HBI: *r_s _=* −0.524, *p* < 0.001	AUROC for “quiescent disease” = 0.864 (95% CI: 0.811-0.916) Sensitivity = 70% Specificity = 90%
**UC**						
Article	Number of patients (*N*)	PROM	Disease activity measure	Correlation/association/predictive accuracy measures	Correlation	Association and predictive accuracy
Bennebroek Evertsz F et al. 2013^35^	149	P-SCCAI	SCCAI CRP PGA of disease activity	Agreement between SCCAI and P-SCCAI was calculated using Spearman’s rho. Agreement between P-SCCAI and SCCAI and between CRP and PGA vs. P-SCCAI to detect remission (SCCAI >5) was calculated by percent agreement and Cohen’s κ. Sensitivity, specificity, PPV, and NPV were calculated from the article data	P-SCCAI vs. SCCAI: *r_s_* = 0.79	P-SCCAI vs. SCCAI: 87% agreement (κ = 0.66) Sensitivity = 91% Specificity = 79% PPV = 94% NPV = 69% P-SCCAI vs. CRP: κ = 0.32, *p *= 0.04 P-SCCAI vs. PGA: κ = 0.73, *p *< 0.001
Bodger K et al. 2014^36^	139	IBD-­Control	SCCAI	Correlation was expressed as Spearman’s *r_s_* values. Negative correlation expected (the higher the IBD-c, the lower the symptoms score). AUROC, sensitivity, specificity, PPV, and NPV to predict “quiescent diseases” pooled for UC and CD	IBD-Control-8 vs. SCCAI: *r_s _*= −0.72, *p *< 0.01 IBD-Control-8 vs. PGA: *r_s_* = −0.67, *p *< 0.01 IBD-Control-VAS vs. SCCAI: *r_s_* = −0.75, *p* < 0.01 IBD-Control-VAS vs. PGA: *r_s_* = −0.65, *p* < 0.01	NA
Clara I et al. 2009^37^	169	MIBDI	PTI	Sensitivity and specificity values to detect activity (PTI ≥4) were provided at 3 time periods (0, 12, and 24 months). PPV and NPV were calculated using the data provided in the study	NA	At 0, 12, and 24 months, respectively: Sensitivity = 84%, 82%, 66% Specificity = 61%, 68%, 61% PPV = 75%, 79%, 68% NPV = 74%, 72%, 59%
Golovics PA et al. 2021^39^	171	PRO-2, pMCS	CRP FCP	Kappa and Chi-2 test for correlation by PROMs-­defined remission and elevated CRP or FCP	k agreement: PRO-2 vs. CRP: κ ∼ 0.2 pMCS vs. CRP: κ ∼ 0.2 PRO-2 vs. FCP: κ = 0.6 pMCS vs. FCP: κ = 0.59	NA
Kamat N et al. 2022^40^	57	IBD-­Control-8, IBD-Control-VAS	SCCAI, FCP	Spearman’s rank correlation coefficient. A negative correlation was expected. AUROC, sensitivity, and specificity to detect remission (SCCAI ≤2)	IBD-Control-8 vs. SCCAI: *r_s _*= −0.59, *p* < 0.001 vs. FCP: *r_s_* = 0.57, *p* < 0.001 IBD-Control-VAS vs. SCCAI: *r_s _*= −0.60, *p* < 0.001 vs. FCP: *r_s _*= −0.40, *p* < 0.001	IBD-Control-8: AUROC = 0.87 (95% CI: 0.78-0.97) Sensitivity = 71.8% Specificity = 88% IBD-Control-VAS: AUROC = 0.83 (95% CI: 0.72-0.94) Sensitivity = 34.4% Specificity = 92% FCP: AUROC: 0.88 (95% CI: 0.77-0.97), *p *< 0.001 Sensitivity = 81.3% Specificity = 88%
Sexton KA et al. 2019^42^	125	IBDSI (long form, LF [38 items])	Nurse-administered PTI	Pearson correlation, AUROC, sensitivity, specificity, PPV, and NPV using PTI ≤5 or PGA	IBDSI vs. PTI: *r *= 0.85, *p* < 0.001 IBDSI vs. PGA: *r *= 0.70, *p *< 0.0001	IBDSI (LF) vs. PTI AUROC: 0.95 (95% CI: 0.92-0.99) Sensitivity: 91% Specificity: 87%
Subramanian S et al. 2016^43^	196	IBD-10 NRS	SCCAI CRP FCP	Spearman’s rank correlation coefficient (Spearman’s rho). AUROC, sensitivity, and specificity were calculated for remission (SCCAI ≤2)	IBD-10 vs. SCCAI: *r_s _*= −0.79, *p* < 0.001	AUROC = 0.94 (95% CI: 0.89-0.97) Sensitivity = 90% (95% CI: 86-94) Sensitivity = 75% (95% CI: 67-82) Patients with CRP <5 mg/dL had a significantly higher IBD-10 score than patients with CRP ≥5 (*p* = 0.002)
Surti B et al. 2013^44^	81	IBD NRS	SCCAI CRP ESR FCP	Pearson correlation coefficient (*r*)	NRS vs. SCCAI: *r* = 0.25, *p* < 0.0001 vs. CRP, ESR, or FCP: no correlation	NA
Tow KE et al. 2019^45^	33	PRO-2-UC	Clinician-verified pMCS	Spearman’s correlation coefficient	PRO-2-UC (paper-based) vs. pMCS: *r_s_* = 0.99, *p* < 0.001 PRO-2-UC (tablet-based) vs. pMCS: *r_s_* = 0.56, *p* < 0.01	NA
Van Deen WK et al. 2016^32^	109	mHI-UC	pMCS	Spearman’s correlation coefficient, ROC analysis to predict disease activity	*r_s_* = 0.72, *p* < 0.0001	AUROC = 0.91 Sensitivity = 73% Specificity = 90%
Vicente Lidón R et al. 2021^46^	71	IBD-Control	SCCAI	Spearman’s correlation coefficient. Sensitivity and specificity for “quiescent disease” (data pooled, separate data for CD and UC were not provided)	*r_s_* = −0.524, *p* < 0.001	AUROC for “quiescent disease” = 0.864 (95% CI: 0.811-0.916) Sensitivity = 70% Specificity = 90%

Some studies analyzed the correlation with disease activity in both CD and UC.

Abbreviations: AP, abdominal pain; AUROC, area under the receiver operating characteristic curve; CD, Crohn’s disease; CDAI, Crohn’s Disease Activity Index; CDEIS, Crohn’s Disease Endoscopic Index of Severity; CI, confidence interval; CRP, C-reactive protein; ESR, erythrocyte sedimentation rate; FCP, fecal calprotectin; GELS, global evaluation of lesion severity; HBI, Harvey-Bradshaw Index; IBD, inflammatory bowel disease; IBD-Control-8, inflammatory bowel disease control-8 items; IBD-Control-VAS, inflammatory bowel disease control-visual analogue scale; IBDQ, Inflammatory Bowel Disease Questionnaire; IBDSI, Inflammatory Bowel Disease Symptom Inventory; MES, Mayo endoscopic subscore; MHBI, modified Harvey-Bradshaw Index; mHI-CD, mobile Health Index for Crohn’s disease; mHI-UC, mobile Health Index for ulcerative colitis; MIAH, Monitor IBD at Home; MIBDI, Manitoba IBD Index; NA, not available; NPV, negative predictive value; NRS, Numerical Rating Scale; PGA, Physician's Global Assessment; p-HBI: patient-based Harvey-Bradshaw Index; pMCS, partial Mayo Clinic Score; PPV, positive predictive value; PRO-2, 2 item patient-reported outcome; PRO-3, 3 item patient-reported outcome; PROM, patient-reported outcome measure; *r*, Pearson correlation coefficient; RB, rectal bleeding; ROC, receiver operating characteristic; *r*_s_, Spearman’s correlation coefficient; SCCAI, Simple Clinical Colitis Activity Index; SES-CD, Simple Endoscopic Score for CD; SF, stool frequency; UC, ulcerative colitis; VAS, visual analogue scale.

### 3.6 Correlation and predictive performance between PROMs and clinical measures of disease activity

Correlations between PROMs and disease activity evaluated by healthcare professionals were moderate-to-strong both in UC and CD in most of the studies (Table 2).^32^^,^^34–37^^,^^40^^,^^42–46^ In CD, *r* values ranged from 0.25 for the correlation between IBD NRS and Harvey-Bradshaw Index^44^ to 0.95 for those between PRO2 and a modified Harvey-Bradshaw Index.^45^ Most correlations were strong (0.50 to 0.90).^32^^,^^34^^,^^36^^,^^42–46^ Sensitivity and specificity values spanned from 70%^46^ to 95%^43^ and from 51%^37^ to 91%,^35^ respectively. AUROCs ranged from 0.86^43^ to 0.93.^42^

In patients with UC, the values for correlations were between 0.25^44^ and 0.99,^45^ with most studies reporting values above 0.50.^32^^,^^34^^,^^36^^,^^40^^,^^42–46^ Sensitivity ranged from 34%^40^ to 91%^35^^,^^42^ and specificity from 61%^37^ to 92%.^40^ AUROCs were reported in 2 studies, with values from 0.83^40^ to 0.95.^42^

## 4. Discussion

Our systematic review shows a poor correlation of PROMs and more objective endoscopic measures of disease activity especially for CD. Correlations were also very poor for laboratory-based activity measures, whereas, as expected, correlations were adequate for some of the clinical activity measures. Although PROMs are valuable tools for capturing the patient’s perspective, their ability to detect inflammatory activity in CD seems limited. For UC, predictive value is better, although most of the predicted value comes from self-reporting rectal bleeding. Finally, our data do not suggest that a particular PROM set might perform much better than the others for detecting endoscopic activity.

Most studies we analyzed had a real-world observational design, underscoring the growing recognition of the value of PROs in understanding and managing IBD beyond controlled clinical trials. The most common symptoms in the identified PROMs were SF, RB, and AP. Some tools also considered extraintestinal/systemic manifestations, general well-being, and emotional/self-perception aspects providing a more comprehensive overview of the patient’s condition. Incorporating these aspects did not seem, however, to improve the ability to detect active inflammation. Interestingly, none of the identified PROMs fully evaluated all relevant signs and symptoms of the diseases. Significant symptoms, such as tenesmus, are absent from all evaluated PROMs, highlighting gaps in the current tools available for IBD assessment.

In CD, correlations between PROMs and endoscopic measures were uniformly poor. For instance, studies assessing the commonly used PRO-2 reported that it was weakly correlated with the SES-CD.^47^ Regarding individual symptoms, the SF item of the MIAH questionnaire showed the highest correlation with SES-CD; however, it was still a modest association.

In UC, correlations were generally stronger than those observed in CD. PROMs, like PRO-2, the MIAH RB item, and the mHI for UC, showed weak-to-moderate correlations with the MES. The rectal bleeding component of MIAH had a stronger correlation with endoscopic findings, suggesting that self-reported bleeding may rather accurately reflect UC disease activity. Indeed, in a meta-analysis of 5 studies, the authors found that most patients with UC who had normal RB and SF subscores had attained endoscopic remission.^48^

Our results agree with prior studies showing that PROMs are not standalone markers of disease activity in IBD. For example, the correlations between PROMs and endoscopic scores in CD were also weak according to Khanna et al.^49^ On the other hand, the study performed by Ma et al. in UC^41^ showed that the correlations of PRO-2 with endoscopic scores were better when determining the value of the RB, which is in line with our findings. This all underlines the importance of the specificity of symptoms in the utility of PROMs.

The reviewed studies also analyzed the correlations between PROMs and laboratory parameters such as CRP and FCP. Neither the IBD-10 nor the HBI showed a good association with high levels of FCP in CD. In the same way, the NRS did not show any correlation with either CRP, ESR, or FCP. In UC, associations were slightly better but still indicated limitations. For instance, IBD-Control-8 and IBD-Control-VAS showed only a moderate correlation with FCP. Previous studies have found similar results, showing that while FCP is a reliable marker for detecting mucosal inflammation, its correlation with PROMs like the IBD-10 and HBI in IBD is generally weak, likely due to the nonspecific nature of the symptoms of IBD that are measured by PROMs.^40^

Regarding the correlation between PROMs and clinically assessed scores, we found heterogenous results, but, as expected, the correlation between different symptom-based scores was acceptable, with some exceptions. In CD, the IBD-10 showed a strong negative correlation with the HBI and the SCCAI indices. In UC, tools like the 6-point Mayo Clinic Score and SCCAI exhibited high sensitivity and specificity in predicting clinical remission measured by SCCAI. Therefore, their alignment with clinical activity indexes is generally better than those with endoscopic measures but can still differ significantly depending on the disease context and specific PROM used.

After closing the review period, a new study reported the correlation between RB, SF, PRO-2, partial Mayo Clinic Score, and SCCAI and endoscopic healing in UC. Correlation ranged from moderate to strong, with no significant differences in predictive accuracy between these measures.^50^ Another investigation recently published in IBD indicated that urgency for bowel movements was consistently associated with disease activity and that fatigue is the most prevalent symptom, irrespective of the disease activity measure used;^51^ Sninsky et al. also supported this finding.^52^ However, it is noteworthy to mention that fecal urgency and fatigue were included in only 5 and 4 out of the 17 PROMs that we identified, respectively. Similarly, Moon et al.^53^ emphasized the importance of considering fecal urgency, tenesmus, mucoid stool, and night-time defecation to improve the prediction of endoscopic mucosal healing status with respect to RB and SF in UC.^53^

The main limitation of this work arises from the challenge of precisely categorizing PROMs based on their intended purpose. Many instruments mix various patient-reported aspects such as general health-related quality of life, disease burden, disability, and disease activity.^54^ Moreover, the considerable heterogeneity observed among the selected studies, derived from differences in study design and comparator selection, complicates the formulation of definitive conclusions based on the results. An additional critical limitation is the inherent bias in patient-reported outcomes, which can be influenced by various factors, including psychological state, socio-economic status, and the patient-clinician relationship. This subjectivity, while valuable in understanding the patient’s perspective, necessitates cautious interpretation of PROMs in isolation from clinical assessments. Furthermore, it should be noted that the quality of conclusions made based on a systematic review largely depends on the biases that may affect the gathering of the original literature, such as the language bias (a type of selection bias) and publication bias toward positive results, which is independent of our methodology. We have included only studies reported in English or Spanish, and have attempted to mitigate possible biases by searching within 3 of the main publicly accessible databases of medical literature, as well as performing manual searches and reviewing the bibliographies of the studies found. We acknowledge that the quality of the available and included studies was generally low-to-moderate. We hope this review will raise awareness about the need to standardize how PROMs are assessed and validated. Finally, the high heterogeneity in the activity measure used as standard, the methods of correlation and prediction estimates, as well as cut-offs used for calculating sensitivity and specificity make it difficult to quantitatively compare between studies. Despite this heterogeneity, the conclusions on the poor correlation between PROMs and endoscopy were consistent, suggesting that the findings of our review are likely to be generalizable.

Nonetheless, our investigation also has some strengths. Our analysis provides a global review of the available validated IBD-specific PROMs, offering valuable insights into their utility and limitations. Moreover, the utilization of a rigorous systematic review methodology, including a detailed literature search and the quality assessment of the included studies, enhances the reliability of our findings.

In conclusion, our review suggests that PROMs cannot substitute objective measures of disease activity in IBD, such as endoscopic and laboratory results. This limitation may be attributed to the fact that IBD symptoms can overlap with those of other gastrointestinal disorders, particularly in CD. However, these results must be considered with caution due to the generally high methodological heterogeneity between studies and low-to-moderate quality of the original studies. Homogenization of methods and standards is warranted for future validation studies. Nonetheless, PROMs represent a valuable complementary instrument to describe disease control comprehensively by capturing the patient’s perspective. Further studies are needed to develop more refined PROMs for IBD, ensuring they capture relevant symptoms and correlate more closely with objective disease measures. Moreover, developing composite indices integrating PROMs with clinical, endoscopic, and laboratory measures could enhance the accuracy and utility of patient-reported data in clinical practice.

## Supplementary Material

jjaf132_Supplementary_Data

## Data Availability

The data underlying this article were obtained from company-sponsored research and cannot be shared publicly due to company policy. The data may be shared upon reasonable request to the authors.
