# Supplementary material for: Patient-reported outcome measures poorly correlate with objective inflammatory bowel disease activity measures: a systematic review
Source: J Crohns Colitis. 2025 Aug 8;19(9):jjaf132. doi: 10.1093/ecco-jcc/jjaf132 (PMC12552099; doi:10.1093/ecco-jcc/jjaf132)
Supplement: jjaf132_Supplementary_Data [file jjaf132_supplementary_data.docx]

Patient-Reported Outcome Measures Poorly Correlate with Objective Inflammatory Bowel Disease Activity Measures: A Systematic Review.

Xavier Calvet^a–c^, Maria Giovanna Ferrario^d^, Vanessa Marfil^d^, Santos Armenteros^e,*^, Manuel Barreiro-de Acosta^f^

**Short title (50/50 characters):** PROMs correlated poorly with IBD disease activity

^a^Gastroenterology Unit, Parc Taulí University Hospital, Parc Taulí Research and Innovation Institute (I3PT-CERCA), Sabadell, Spain

^b^Department of Medicine, Autonomous University of Barcelona, Bellaterra, Spain

^c^CIBERehd, Carlos III Health Institute, Madrid, Spain

^d^Medical Statistics Consulting SL, Valencia, Spain

^e^Galapagos Biopharma Spain SL, Madrid, Spain

^f^Inflammatory Bowel Disease Unit, University Clinical Hospital of Santiago de Compostela, Santiago de Compostela, Spain

*Former association; employee of Alfasigma S.p.A. at the time of publication.

# **Supplementary Material**

Supplementary Table 1. Search terms

| **Term** | **Synonyms** |
| --- | --- |
| IBD | (“Inflammatory bowel diseases”[MeSH] “Crohn disease”[MeSH] OR “Colitis, Ulcerative”[MeSH] OR (Inflammatory bowel disease) OR IBD OR (Crohn disease) OR (ulcerative colitis)) |
| PROM | ((patient-reported outcome* measure*) OR “Patient Reported Outcome Measures”[MeSH] OR PROM*) |
| Instrument | (score OR questionnaire OR index OR scale OR instrument OR (assessment tool)) |
| Signs and symptoms | ((abdominal pain) OR “abdominal pain”[MeSH] OR tenesmus OR fatigue OR fatigue[MeSH] OR (rectal bleeding) OR (gastrointestinal hemorrhage) OR (deposition frequency) OR (fecal incontinence) OR “fecal incontinence”[MeSH] OR anxiety OR depression) |
| Correlation with clinical outcomes | (((Remission) OR (disease activity) OR (disease control)) OR ((fecal calprotectin) OR FCP OR (C-Reactive Protein) OR CRP OR endoscopy OR (Erythrocyte sedimentation rate) OR ESR) OR ((physician global assessment) OR PGA OR (Crohn’s Disease Activity Index) OR CDAI OR (Harvey-Bradshaw Index) OR HBI OR (Simple Clinical Colitis Activity Index) OR SCCAI OR (Mayo score)))) |
| Self-reported | (Patient-reported OR Self-reported) |

Supplementary Table 2. Search strategies

| **PubMed** | **Scopus** | **Web of Science** | **Google Scholar** |
| --- | --- | --- | --- |
| ("Inflammatory bowel diseases"[MeSH] OR "Crohn disease"[MeSH] OR "Colitis, Ulcerative"[MeSH] OR "Inflammatory bowel disease" OR IBD OR "Crohn disease" OR "ulcerative colitis") AND ("patient-reported outcome? Measure?"[tiab] OR "Patient Reported Outcome Measures"[MeSH] OR PROM?[tiab] OR ((patient-reported OR self-reported) AND (score[tiab] OR questionnaire[tiab] OR index[tiab] OR scale[tiab] OR instrument[tiab] OR "assessment tool"[tiab]))) AND (Remission OR (disease activity) OR (disease control) OR (fecal calprotectin) OR FCP OR (C-Reactive Protein) OR CRP OR endoscopy OR (Erythrocyte sedimentation rate) OR ESR OR (physician global assessment) OR PGA OR (Crohn’s Disease Activity Index) OR CDAI OR (Harvey-Bradshaw Index) OR HBI OR (Simple Clinical Colitis Activity Index) OR SCCAI OR (Mayo Score)) | ((Inflammatory bowel disease) OR IBD OR (Crohn disease) OR (ulcerative colitis)) AND ("patient-reported outcome? Measure?" OR PROM? OR ((patient-reported OR self-reported) AND (score OR questionnaire OR index OR scale OR instrument OR (assessment tool)))) AND (Remission OR (disease activity) OR (disease control) OR (fecal calprotectin) OR FCP OR (C-Reactive Protein) OR CRP OR endoscopy OR (Erythrocyte sedimentation rate) OR ESR OR (physician global assessment) OR PGA OR (Crohn’s Disease Activity Index) OR CDAI OR (Harvey-Bradshaw Index) OR HBI OR (Simple Clinical Colitis Activity Index) OR SCCAI OR (Mayo Score)) | ((Inflammatory bowel disease) OR IBD OR (Crohn disease) OR (ulcerative colitis)) AND ("patient-reported outcome? Measure?" OR PROM? OR ((patient-reported OR self-reported) AND (score OR questionnaire OR index OR scale OR instrument OR (assessment tool)))) AND (Remission OR (disease activity) OR (disease control) OR (fecal calprotectin) OR FCP OR (C-Reactive Protein) OR CRP OR endoscopy OR (Erythrocyte sedimentation rate) OR ESR OR (physician global assessment) OR PGA OR (Crohn’s Disease Activity Index) OR CDAI OR (Harvey-Bradshaw Index) OR HBI OR (Simple Clinical Colitis Activity Index) OR SCCAI OR (Mayo Score)) | "IBD", "Patient-Reported Outcomes", "PROM", "disease activity", "España", "enfermedad inflamatoria intestinal", "medición de actividad", "resultados reportados por el paciente", "actividad clínica", "medición de la actividad de la enfermedad". |

Supplementary Table 3. Reasons for excluding articles

| **Article** | **Title** | **Reason for exclusion** |
| --- | --- | --- |
| Abdalla MI *et al.* 2017^1^ | Prevalence and impact of inflammatory bowel disease-irritable bowel syndrome on patient-reported outcomes in CCFA partners | Clinical measure not self-reported |
| Abraham B *et al.* 2022^2^ | Impact of infliximab-dyyb (infliximab biosimilar) on clinical and patient-reported outcomes: 1-year follow-up results from an observational real-world study among patients with inflammatory bowel disease in the US and Canada (the ONWARD study) | Clinical measure not self-reported |
| Adegbola SO *et al.* 2018^3^ | Symptom Amelioration in Crohn's perianal fistulas using video-assisted anal fistula treatment (VAAFT) | PROM not specific to IBD |
| Armuzzi A *et al.* 2020^4^ | The association between disease activity and patient-reported outcomes in patients with moderate-to-severe ulcerative colitis in the United States and Europe | Clinical measure not self-reported |
| Artom M *et al.* 2017^5^ | The contribution of clinical and psychosocial factors to fatigue in 182 patients with inflammatory bowel disease: a cross-sectional study | PROM not an outcome |
| Arvanitis M *et al.* 2021^6^ | Transition readiness not associated with measures of health in youth with IBD | Clinical measure not self-reported |
| Avery P. 2021^7^ | Using e-health tools and PROMs to support self-management in patients with inflammatory bowel disease | Clinical measure not self-reported |
| Banovic I *et al.* 2020^8^ | Toward further understanding of Crohn's disease-related fatigue: the role of depression and emotional processing | Clinical measure not self-reported |
| Bieliński M *et al.* 2018^9^ | Affective temperament in inflammatory bowel diseases: another brick in the wall of differentiation | Clinical measure not self-reported |
| Byrne G *et al.* 2017^10^ | Prevalence of Anxiety and Depression in Patients with Inflammatory Bowel Disease | Clinical measure not self-reported |
| Calixto RP *et al.* 2018^11^ | Inflammatory bowel disease: impact on scores of quality of life, depression and anxiety in patients attending a tertiary care center in Brazil | Clinical measure not self-reported |
| Calvet X *et al.* 2018^12^ | Patients’ perceptions of the impact of ulcerative colitis on social and professional life: results from the UC-LIFE survey of outpatient clinics in Spain | PROM not an outcome |
| Calvet X *et al.* 2021^13^ | Patient-evaluated quality of care is related to better inflammatory bowel disease outcomes: the IQCARO II project | PROM not an outcome |
| Casellas F *et al.* 2021^13^ | Factors associated with quality of care in inflammatory bowel diseases: a view from patient's side using the IQCARO quality of care decalogue | PROM not an outcome |
| Chao CY *et al.* 2019^14^ | Maladaptive coping, low self-efficacy and disease activity are associated with poorer patient-reported outcomes in inflammatory bowel disease | PROM not an outcome |
| Chmiel C *et al.* 2019^15^ | Can the CalproQuest predict a positive calprotectin test? a prospective diagnostic study | PROM not validated |
| Christiansen LK *et al.* 2019^16^ | Health-related quality of life in inflammatory bowel disease in a Danish population-based inception cohort | PROM not an outcome |
| Claar RL *et al.* 2017^17^ | Psychological distress and quality of life in pediatric Crohn disease: impact of pain and disease state | Clinical measure not self-reported |
| Coenen S *et al.* 2019^18^ | Short health scale: a valid and reliable measure of quality of life in Dutch speaking patients with inflammatory bowel disease | PROM validation in a language other than English or Spanish |
| Coenen S *et al.* 2020^19^ | Development and feasibility of a telemonitoring tool with full integration in the electronic medical record: a proof-of-concept study for patients with inflammatory bowel disease in remission on biological therapy | PROM not an outcome |
| Cushman G *et al.* 2021^20^ | Age, disease symptoms, and depression are associated with body image dissatisfaction in newly diagnosed pediatric inflammatory bowel disease | Clinical measure not self-reported |
| de Jong ME *et al.* 2021^21^ | Cross-cultural translation and validation of the IBD-control questionnaire in The Netherlands: a patient-reported outcome measure in inflammatory bowel disease | PROM validation in a language other than English or Spanish |
| Dibley L *et al.* 2021^22^ | Clinician administered and self-report survey both effective for identifying fecal incontinence in patients with inflammatory bowel disease | Clinical measure not self-reported |
| Dubinsky MC *et al.* 2021^23^ | Ulcerative colitis narrative global survey findings: the impact of living with ulcerative colitis-patients' and physicians' view | PROM not specific to IBD |
| Dubinsky MC *et al.* 2022^24^ | Incorporating patient experience into drug development for ulcerative colitis: development of the urgency numeric rating scale, a patient-reported outcome measure to assess bowel urgency in adults | PROM not an outcome |
| Dulai PS *et al.* 2020^25^ | Prevalence of endoscopic improvement and remission according to patient-reported outcomes in ulcerative colitis | PROM not an outcome |
| Dulai PS *et al.* 2020^26^ | Development of the symptoms and impacts questionnaire for Crohn's disease and ulcerative colitis | PROM not validated |
| Engel K *et al.* 2021^27^ | Newly diagnosed patients with inflammatory bowel disease: the relationship between perceived psychological support, health-related quality of life, and disease activity | Clinical measure not self-reported |
| Frigstad SO *et al.* 2019^28^ | Pain severity and vitamin d deficiency in ibd patients | Clinical measure not self-reported |
| Ghosh S *et al.* 2019^29^ | Healthcare providers underestimate patients’ glucocorticoid use in crohn’s disease | PROM not an outcome |
| Gollifer RM *et al.* 2018^30^ | Relationship between MRI quantified small bowel motility and abdominal symptoms in Crohn's disease patients-a validation study | Clinical measure not self-reported |
| Gong W *et al.* 2021^31^ | JINLING (Judicious INdex of Luminal INflammation Grade) score, an effective indicator to assess inflammation severity in Crohn's disease | Clinical measure not self-reported |
| Gorrepati VS *et al.* 2020^32^ | Abdominal pain and anxious or depressed state are independently associated with weight loss in inflammatory bowel disease | Clinical measure not self-reported |
| Gracie DJ *et al.* 2018^33^ | Longitudinal impact of IBS-type symptoms on disease activity, healthcare utilization, psychological health, and quality of life in inflammatory bowel disease | Clinical measure not self-reported |
| Grant A *et al.* 2021^34^ | Assessing disease activity using the pediatric Crohn's disease activity index: Can we use subjective or objective parameters alone? | Clinical measure not self-reported |
| Halloran J *et al.* 2021^35^ | Psychosocial burden of inflammatory bowel disease in adolescents and young adults | PROM not an outcome |
| Hisamatsu T *et al.* 2021^36^ | Long-term safety and effectiveness of adalimumab in Japanese patients with Crohn's disease: 3-year results from a real-world study | Clinical measure not self-reported |
| Hoekman DR *et al.* 2022^37^ | Hypnotherapy for irritable bowel syndrome-type symptoms in patients with quiescent inflammatory bowel disease: a randomized, controlled trial | PROM not specific to IBD |
| Hueppe A 2018^38^ | Validation of the "German Inflammatory Bowel Disease Activity Index (GIBDI)": an instrument for patient-based disease activity assessment in Crohn's disease and ulcerative colitis | PROM validation in a language other than English or Spanish |
| Irving P *et al.* 2018^39^ | A randomized, double-blind, placebo-controlled, parallel-group, pilot study of cannabidiol-rich botanical extract in the symptomatic treatment of Ulcerative colitis | PROM not an outcome |
| Janssen L *et al.* 2018^40^ | Control Crohn Safe with episodic adalimumab monotherapy as first-line treatment study (CoCroS): study protocol for a randomised controlled trial | PROM not validated |
| Jain A *et al.* 2018^41^ | Impact of obesity on disease activity and patient-reported outcomes measurement information system (PROMIS) in inflammatory bowel diseases | PROM not specific to IBD |
| Jorgessen KK *et al.* 2020^42^ | Efficacy and safety of CT-P13 in inflammatory bowel disease after switching from originator infliximab: exploratory analyses from the NOR-SWITCH main and extension Trials | PROM not specific to IBD |
| Kaazan P *et al.* 2021^43,44^ | Assessing effectiveness and patient perceptions of a novel electronic medical record for the management of inflammatory bowel disease | PROM not an outcome |
| Kaimakliotis P *et al.* 2021 | Targeted housestaff intervention reduces opioid use without worsening patient-reported pain scores and improves outcomes among patients with IBD: the “IBD pain ladder” | PROM not specific to IBD |
| Kamal N *et al.* 2021^45^ | Fecal incontinence in inflammatory bowel disease | Clinical measure not self-reported |
| Kamp KJ *et al.* 2021 | Dietary patterns, beliefs and behaviours among individuals with inflammatory bowel disease: a cross-sectional study | PROM not specific to IBD |
| Kani HT *et al.* 2020^46^ | Validation and reliability of the Turkish version of the inflammatory bowel disease questionnaire for ulcerative colitis and Crohn's disease | PROM validation in a language other than English or Spanish |
| Keller *et al.* 2021^47^ | Quality of life in inflammatory bowel diseases: it is not all about the bowel | PROM not an outcome |
| Khera AJ *et al.* 2019^48^ | Gut-directed pelvic floor behavioral treatment for fecal incontinence and constipation in patients with inflammatory bowel disease | PROM not specific to IBD |
| Kim A *et al.* 2018^49^ | Developing a standard set of patient-centred outcomes for inflammatory bowel disease-an international, cross-disciplinary consensus | PROM not validated |
| Kim B *et al.* 2021^50^ | Physical activity and quality of life of patients with inflammatory bowel disease | PROM not an outcome |
| Kim ES *et al.* 2017^51^ | Development of a web-based, self-reporting symptom diary for Crohn's disease, and its correlation with the Crohn's Disease Activity Index | PROM not validated |
| Kim ES *et al.* 2018^52^ | Disease activity patterns recorded using a mobile monitoring system are associated with clinical outcomes of patients with Crohn's disease | PROM not validated |
| Koch AK *et al.* 2019^53^ | Perceived stress mediates the effect of yoga on quality of life and disease activity in ulcerative colitis. Secondary analysis of a randomized controlled trial | Clinical measure not self-reported |
| Kochar B *et al.* 2018^54^ | Inflammatory bowel disease is similar in patients with older onset and younger onset | Clinical measure not self-reported |
| Kruis W *et al.* 2022^55^ | Novel budesonide suppository and standard budesonide rectal foam induce high rates of clinical remission and mucosal healing in active ulcerative proctitis, a randomised, controlled, non-inferiority trial | Clinical measure not self-reported |
| Kumar A *et al.* 2020^56^ | Patient-preferences favoring treatment discontinuation are reduced with vedolizumab and ustekinumab compared with tnf antagonists in inflammatory bowel disease | PROM not validated |
| Lahat A *et al.* 2020^57^ | Change in bowel habits during menstruation: are IBD patients different? | PROM not validated |
| Larussa T *et al*. 2020^58^ | The reality of patient-reported outcomes of health-related quality of life in an Italian cohort of patients with inflammatory bowel disease: results from a cross-sectional study | PROM not an outcome |
| LeBerre C *et al.* 2020^59^ | Protocol of a multicentric prospective cohort study for the VALIDation of the IBD-disk instrument for assessing disability in inflammatory bowel diseases: the VALIDate study | PROM not validated |
| Lee A *et al.* 2021^60^ | Clinical hypnosis in pediatric Crohn's disease: a randomized controlled pilot study | Clinical measure not self-reported |
| Li J *et al.* 2022^61^ | Impact of the SARS-CoV-2 delta variant on the psychological states and health-related quality of life in patients with Crohn's disease | PROM not an outcome |
| Lightner AL *et al.* 2022^62^ | A phase IB/IIA study of remestemcel-L, an allogeneic bone marrow derived mesenchymal stem cell product, for the treatment of medically refractory ulcerative colitis: An interim analysis | Clinical measure not self-reported |
| Lo B *et al.* 2018^63^ | Disease activity, steroid use and extraintestinal manifestation are associated with increased disability in patients with inflammatory bowel disease using the inflammatory bowel disease disability index: A cross-sectional multicentre cohort study | PROM validation in a language other than English or Spanish |
| Loftus EV *et al.* 2019^64^ | Adalimumab effectiveness up to six years in adalimumab-naïve patients with Crohn's disease: results of the PYRAMID registry | Clinical measure not self-reported |
| Ludvigsson JF *et al*. 2019^65^ | Swedish Inflammatory Bowel Disease Register (SWIBREG) - a nationwide quality register | PROM not specific to IBD |
| Marcovitch L *et al.* 2017^66^ | Item generation and reduction toward developing a patient-reported outcome for pediatric Ulcerative colitis (TUMMY-UC) | PROM not validated |
| Marcovivitch L *et al.* 2021^67^ | Agreement on symptoms between children with ulcerative colitis and their caregivers: towards developing the TUMMY-UC | PROM not validated |
| Marinelli C *et al.* 2019^68^ | Factors influencing disability and quality of life during treatment: a cross-sectional study on IBD patients | PROM not an outcome |
| Marrie RA *et al.* 2021^69^ | The relationship between symptoms of depression and anxiety and disease activity in IBD over time | PROM not specific to IBD |
| Masoodi M *et al.* 2018^70^ | The efficacy of curcuminoids in improvement of ulcerative colitis symptoms and patients' self-reported well-being: a randomized double-blind controlled trial | Clinical measure not self-reported |
| Melmed GY *et al.* 2021^71^ | Quality of care program reduces unplanned health care utilization in patients with inflammatory bowel disease | PROM not an outcome |
| Molander P *et al.* 2019^72^ | Impact of ulcerative colitis on patients’ lives: results of the Finnish extension of a global ulcerative colitis narrative survey | PROM not validated |
| Moon JR *et al.* 2020^73^ | Unmet psychosocial needs of patients with newly diagnosed ulcerative colitis: results from the nationwide prospective cohort study in Korea | PROM not specific to IBD |
| Moum KM *et al.* 2021^74^ | Patients with inflammatory bowel disease on immunosuppressive drugs: perspectives' on COVID-19 and health care service during the pandemic | PROM not specific to IBD |
| Murphy LK *et al.* 2020^75^ | Biopsychosocial correlates of presence and intensity of pain in adolescents with inflammatory bowel disease | PROM not an outcome |
| Naegeli AN *et al.* 2021^76^ | Full, partial, and modified permutations of the mayo score: characterizing clinical and patient-reported outcomes in ulcerative colitis patients | PROM not an outcome |
| Nguyen NH *et al.* 2022^77^ | Patient-reported outcomes and risk of hospitalization and readmission in patients with inflammatory bowel diseases | PROM not specific to IBD |
| Nishida Y et al 2021^78^ | Impact of the lockdown due to the COVID-19 pandemic on patients with inflammatory bowel disease | PROM not validated |
| Norton C *et al.* 2021^79^ | Supported online self-management versus care as usual for symptoms of fatigue, pain and urgency/incontinence in adults with inflammatory bowel disease (IBD-BOOST): study protocol for a randomised controlled trial | PROM not validated |
| Olden KW *et al.* 2019^80^ | Alosetron versus traditional pharmacotherapy in clinical practice: effects on resource use, health-related quality of life, safety and symptom improvement in women with severe diarrhea-predominant irritable bowel syndrome | PROM not specific to IBD |
| Ostromohov G *et al.* 2022^81^ | Assessment of patients' understanding of inflammatory bowel diseases: development and validation of a questionnaire | PROM not an outcome |
| Omori T *et al.* 2021^82^ | Real-world safety and efficacy of twice-daily budesonide 2-mg foam in patients with ulcerative colitis: interim analysis of post-marketing surveillance | Clinical measure not self-reported |
| Opheim R *et al.* 2020^83^ | Self-esteem in patients with inflammatory bowel disease | Clinical measure not self-reported |
| Otto-Sobotka F *et al.* 2019^84^ | Modeling determinants of satisfaction with health care in youth with inflammatory bowel disease part 2: semiparametric distributional regression | PROM not an outcome |
| Panés J *et al.* 2018^85^ | Tofacitinib in patients with ulcerative colitis: health-related quality of life in phase 3 randomised controlled induction and maintenance studies | PROM not an outcome |
| Panés J *et al.* 2019^86^ | Long-term safety and tolerability of oral tofacitinib in patients with Crohn’s disease: results from a phase 2, open-label, 48-week extension study | PROM not an outcome |
| Paramsothy S *et al.* 2017^87^ | Multidonor intensive faecal microbiota transplantation for active ulcerative colitis: a randomised placebo-controlled trial | PROM not an outcome |
| Parra Izquierdo V *et al.* 2021^88^ | P056 Real life experience with the use of tofacitinib in ulcerative colitis in Colombia: case series | Clinical measure not self-reported |
| Parra RS *et al.* 2019^89^ | Quality of life, work productivity impairment and healthcare resources in inflammatory bowel diseases in Brazil | PROM not an outcome |
| Pittet V *et al*. 2017^90^ | Patient self-reported concerns in inflammatory bowel diseases: A gender-specific subjective quality-of-life indicator | PROM not validated |
| Pittet V *et al*. 2018^91^ | Patient-reported healthcare expectations in inflammatory bowel diseases | PROM not validated |
| Pittet V *et al*. 2019^92^ | Differences in outcomes reported by patients with inflammatory bowel diseases vs their health care professionals | PROM not an outcome |
| Probert CS *et al.* 2018^93^ | Golimumab induction and maintenance for moderate to severe ulcerative colitis: results from GO-COLITIS (Golimumab: a phase 4, UK, open label, single arm study on its utilization and impact in ulcerative Colitis) | PROM not an outcome |
| Raffals LE *et al.* 2022^94^ | The development and initial findings of a study of a prospective adult research cohort with inflammatory bowel disease (SPARC IBD) | Clinical measure not self-reported |
| Ramos A *et al.* 2018^95^ | Validation of a self-reported work disability questionnaire for ulcerative colitis | Clinical measure not self-reported |
| Rassart J *et al.* 2022^96^ | Illness identity in inflammatory bowel disease | PROM not an outcome |
| Regev S *et al.* 2022^97^ | Normative data for the brief symptom inventory for patients with Crohn's disease | PROM not an outcome |
| Ricci L *et al.* 2020^98^ | Flare-IBD: development and validation of a questionnaire based on patients' messages on an internet forum for early detection of flare in inflammatory bowel disease: study protocol | PROM not an outcome |
| Sacks OA *et al.* 2021^99^ | Poor baseline health of IBD patients at presentation to a surgeon: results from a patient-reported outcomes database | PROM not specific to IBD |
| Sahnan K *et al.* 2019 | Developing a core outcome set for fistulising perianal Crohn's disease | Clinical measure not self-reported |
| Sands BE *et al.* 2018^100^ | The effects of ustekinumab on health-related quality of life in patients with moderate to severe Crohn's disease | Clinical measure not self-reported |
| Sattoe JNT *et al.* 2020^101^ | Value of an outpatient transition clinic for young people with inflammatory bowel disease: a mixed-methods evaluation | PROM not an outcome |
| Schäfer SK *et al.* 2017^102^ | Design and validation of a German version of the GSRS-IBS - an analysis of its psychometric quality and factorial structure | PROM validation in a language other than English or Spanish |
| Sekhri S *et al.* 2021^103^ | Serum trough levels of infliximab are not associated with peripheral arthralgia activity in patients with inflammatory bowel disease | Clinical measure not self-reported |
| Shah R *et al.* 2021^104^ | Telehealth model of care for outpatient inflammatory bowel disease care in the setting of the COVID-19 pandemic | PROM not an outcome |
| Shaw AL *et al.* 2017^105^ | Impact of serum-derived bovine immunoglobulin/protein isolate therapy on irritable bowel syndrome and inflammatory bowel disease: a survey of patient perspective | PROM not validated |
| Shih S *et al.* 2021^106^ | Temperament and Health-Related Quality of Life in Newly Diagnosed Pediatric Inflammatory Bowel Disease | Clinical measure not self-reported |
| Simsek M *et al.* 2018 | Patient-Reported Experiences with a Relicensed Generic: Thioguanine for the Treatment of Inflammatory Bowel Diseases | PROM not an outcome |
| Smyth M *et al.* 2021^107^ | Cross-Sectional Analysis of Quality of Life in Pediatric Patients with Inflammatory Bowel Disease in British Columbia, Canada | PROM not an outcome |
| Spagnuolo R *et al.* 2020^108^ | COVID-19 and inflammatory bowel disease: Patient knowledge and perceptions in a single center survey | PROM not an outcome |
| Stawowczyk E *et al.* 2020^109^ | Productivity Loss Among Parents of Children With Inflammatory Bowel Diseases in Relation to Disease Activity and Patient's Quality of Life | Clinical measure not self-reported |
| Svolos V *et al.* 2017^110^ | Dietary treatment of Crohn's disease: perceptions of families with children treated by exclusive enteral nutrition, a questionnaire survey | PROM not an outcome |
| Taft TH *et al.* 2022^111^ | Posttraumatic Stress in Patients With Inflammatory Bowel Disease: Prevalence and Relationships to Patient-Reported Outcomes | PROM not an outcome |
| Taxonera C *et al.* 2021^112^ | Preferences and satisfaction of IBD patients after switching from adalimumab 40 mg weekly to 80 mg every other week given as a single injection: the ADASCAL study | PROM not an outcome |
| Teich N *et al.* 2021^113^ | Novel Approaches Identifying Relevant Patient-Reported Outcomes in Patients With Inflammatory Bowel Diseases-LISTEN | Clinical measure not self-reported |
| Teich N *et al.* 2021^114^ | Effect of Originator Infliximab Treatment on Disease-Related Hospitalizations, Work Productivity and Activity Impairment, and Health Resource Utilization in Patients with Crohn's Disease in a Real-Life Setting: Results of a Prospective Multicenter Study in Germany | Clinical measure not self-reported |
| Thomas PWA *et al.* 2022^115^ | Impact of Biological Therapies and Tofacitinib on Real-world Work Impairment in Inflammatory Bowel Disease Patients: A Prospective Study | Clinical measure not self-reported |
| Tiankanon K *et al.* 2021^116^ | Burden of Inflammatory Bowel Disease on Patient Mood, Fatigue, Work, and Health-Related Quality of Life in Thailand: A Case-Control Study | PROM not an outcome |
| Timmer A *et al.* 2017^117^ | Current health status and medical therapy of patients with pediatric-onset inflammatory bowel disease: a survey-based analysis on 1280 patients aged 10-25 years focusing on differences by age of onset | PROM not an outcome |
| Tormey LK *et al.* 2019^118^ | Limited Health Literacy Is Associated With Worse Patient-Reported Outcomes in Inflammatory Bowel Disease | Clinical measure not self-reported |
| Trindade IA *et al.* 2017^119^ | An 18-month study of the effects of IBD symptomatology and emotion regulation on depressed mood | PROM not validated |
| Trivedi I *et al.* 2019^120^ | Patient Understanding of "Flare" and "Remission" of Inflammatory Bowel Disease | PROM not validated |
| Tsuda S *et al.* 2018^121^ | Patient self-reported symptoms using visual analog scales are useful to estimate endoscopic activity in ulcerative colitis | PROM not validated |
| Vollebregt PF *et al.* 2017^122^ | Validation of Risk Factors for Fecal Incontinence in Patients With Crohn's Disease | PROM not specific to IBD |
| Watanabe C *et al.* 2021^123^ | Non-adherence to Medications in Pregnant Ulcerative Colitis Patients Contributes to Disease Flares and Adverse Pregnancy Outcomes | Clinical measure not self-reported |
| Wei S-C *et al.* 2018^124^ | Experience of patients with inflammatory bowel disease in using a home fecal calprotectin test as an objective reported outcome for self-monitoring | Clinical measure not self-reported |
| Weizman AV *et al.* 2021^125^ | Providing Hospitalized Ulcerative Colitis Patients With Practice Guidelines Improves Patient-Reported Outcomes | PROM not an outcome |
| Wiestler M *et al.* 2019^126^ | Quality of Life Is Associated With Wearable-Based Physical Activity in Patients With Inflammatory Bowel Disease: A Prospective, Observational Study | Clinical measure not self-reported |
| Williams AJ *et al.* 2021^127^ | Australia IBD Microbiome (AIM) Study: protocol for a multicentre longitudinal prospective cohort study | Clinical measure not self-reported |
| Willian MK *et al.* 2018^128^ | Changes in health-related quality of life and work-related outcomes for patients with mild-to-moderate ulcerative colitis receiving short-term and long-term treatment with multimatrix mesalamine: a prospective, open-label study | Clinical measure not self-reported |
| Wirth MD *et al.* 2020^129^ | The dietary inflammatory index is associated with gastrointestinal infection symptoms in the national health and nutrition examination survey | PROM not an outcome |
| Xu J *et al.* 2021^130^ | Correlation Between Sleep, Life, Mood, and Diet and Severity of Inflammatory Bowel Disease in China: A Retrospective Study | Clinical measure not self-reported |
| Yamabe K *et al.* 2019^131^ | Health-related quality of life outcomes and economic burden of inflammatory bowel disease in Japan | PROM not an outcome |
| Yan X *et al.* 2020^132^ | Assessment of patient-centered outcomes (PROs) in inflammatory bowel disease (IBD): a multicenter survey preceding a cross-disciplinary (functional) consensus | PROM not validated |
| Yu M *et al.* 2020^133^ | Questionnaire assessment helps the self-management of patients with inflammatory bowel disease during the outbreak of Coronavirus Disease 2019 | PROM not validated |
| Zhang JJ *et al.* 2020^134^ | Development of a validated Chinese version of the inflammatory bowel disease disability index | PROM not an outcome |
| Zickgraf HF *et al.* 2022^135^ | "If I could survive without eating, it would be a huge relief": Development and initial validation of the Fear of Food Questionnaire. Appetite | PROM not an outcome |

**References**

1. Abdalla MI, Sandler RS, Kappelman MD, et al. Prevalence and Impact of Inflammatory Bowel Disease-Irritable Bowel Syndrome on Patient-reported Outcomes in CCFA Partners. *Inflammatory Bowel Diseases*. 2017 2017;23(2):325-331. doi:doi:

2. Abraham B, Eksteen B, Nedd K, et al. Impact of Infliximab-dyyb (Infliximab Biosimilar) on Clinical and Patient-Reported Outcomes: 1-Year Follow-up Results from an Observational Real-World Study Among Patients with Inflammatory Bowel Disease in the US and Canada (the ONWARD Study). *Adv Ther*. 2022 2022;39(5):2109-2127. doi:doi:

3. Adegbola SO, Sahnan K, Tozer PJ, et al. Symptom Amelioration in Crohn's Perianal Fistulas Using Video-Assisted Anal Fistula Treatment (VAAFT). *J Crohns Colitis*. 2018 2018;12(9):1067-1072. doi:doi:

4. Armuzzi A, Tarallo M, Lucas J, et al. The association between disease activity and patient-reported outcomes in patients with moderate-to-severe ulcerative colitis in the United States and Europe. *BMC Gastroenterol*. 2020 2020;20(1):18. doi:doi:

5. Artom M, Czuber-Dochan W, Sturt J, Murrells T, Norton C. The contribution of clinical and psychosocial factors to fatigue in 182 patients with inflammatory bowel disease: a cross-sectional study. *Aliment Pharmacol Ther*. 2017 2017;45(3):403-416. doi:doi:

6. Arvanitis M, Hart LC, DeWalt DA, et al. Transition Readiness Not Associated With Measures of Health in Youth With IBD. *Inflammatory Bowel Diseases*. 2021;27(1):49-57.

7. Avery P. Using e-health tools and PROMs to support self-management in patients with inflammatory bowel disease. *Br J Nurs*. 2021 2021;30(7):394-402. doi:doi:

8. Banovic I, Montreuil L, Derrey-Bunel M, et al. Toward Further Understanding of Crohn's Disease-Related Fatigue: The Role of Depression and Emotional Processing. *Frontiers in Psychology*. 2020 2020;11doi:doi:

9. Bieliński M, Lesiewska N, Bielińska J, et al. Affective temperament in inflammatory bowel diseases: Another brick in the wall of differentiation. *PLoS One*. 2018 2018;13(11):e0205606. doi:doi:

10. Byrne G, Rosenfeld G, Leung Y, et al. Prevalence of Anxiety and Depression in Patients with Inflammatory Bowel Disease. *Can J Gastroenterol Hepatol*. 2017 2017;2017:6496727. doi:doi:

11. Calixto RP, Flores C, Francesconi CF. INFLAMMATORY BOWEL DISEASE: IMPACT ON SCORES OF QUALITY OF LIFE, DEPRESSION AND ANXIETY IN PATIENTS ATTENDING A TERTIARY CARE CENTER IN BRAZIL. *Arq Gastroenterol*. 2018 2018;55(3):202-207. doi:doi:

12. Calvet X, Argüelles-Arias F, López-Sanromán A, et al. Patients’ perceptions of the impact of ulcerative colitis on social and professional life: Results from the UC-LIFE survey of outpatient clinics in Spain. *Patient Preference and Adherence*. 2018 2018;12:1815-1823. doi:doi:

13. Calvet X, Casellas F, Saldana R, et al. Patient-Evaluated Quality of Care is Related to Better Inflammatory Bowel Disease Outcomes: The IQCARO II Project. *Patient-Patient Centered Outcomes Research*. 2021 2021;14(5):625-634. doi:doi:

14. Chao C-Y, Lemieux C, Restellini S, et al. Maladaptive coping, low self-efficacy and disease activity are associated with poorer patient-reported outcomes in inflammatory bowel disease. *Saudi Journal of Gastroenterology*. 2019 2019;25(3):159-+. doi:doi:

15. Chmiel C, Senn O, Hasler S, et al. Can the CalproQuest predict a positive Calprotectin test? A prospective diagnostic study. *PLoS One*. 2019 2019;14(11):e0224961. doi:doi:

16. Christiansen LK, Lo B, Bendtsen F, Vind I, Vester-Andersen MK, Burisch J. Health-related quality of life in inflammatory bowel disease in a Danish population-based inception cohort. *United European Gastroenterol J*. 2019 2019;7(7):942-954. doi:doi:

17. Claar RL, van Tilburg MAL, Abdullah B, et al. Psychological Distress and Quality of Life in Pediatric Crohn Disease: Impact of Pain and Disease State. *J Pediatr Gastroenterol Nutr*. 2017;65(4):420-424.

18. Coenen S, Weyts E, Geens P, et al. Short Health Scale: a valid and reliable measure of quality of life in Dutch speaking patients with inflammatory bowel disease. *Scand J Gastroenterol*. 2019 2019;54(5):592-596. doi:doi:

19. Coenen S, Nijns E, Weyts E, et al. Development and feasibility of a telemonitoring tool with full integration in the electronic medical record: a proof of concept study for patients with inflammatory bowel disease in remission on biological therapy. *Scand J Gastroenterol*. 2020 2020;55(3):287-293. doi:doi:

20. Cushman G, Stolz MG, Shih S, et al. Age, Disease Symptoms, and Depression are Associated With Body Image Dissatisfaction in Newly Diagnosed Pediatric Inflammatory Bowel Disease. *J Pediatr Gastroenterol Nutr*. 2021;72(3):e57-e62.

21. de Jong MJ, Roosen D, Degens J, et al. Development and Validation of a Patient-reported Score to Screen for Mucosal Inflammation in Inflammatory Bowel Disease. *J Crohns Colitis*. Apr 26 2019;13(5):555-563. doi:10.1093/ecco-jcc/jjy196

22. Dibley L, Hart A, Duncan J, et al. Clinician Administered and Self-Report Survey Both Effective for Identifying Fecal Incontinence in Patients with Inflammatory Bowel Disease. *Digestive Diseases and Sciences*. 2021 2021;66(6):2024-2031. doi:doi:

23. Dubinsky MC, Watanabe K, Molander P, et al. Ulcerative Colitis Narrative Global Survey Findings: The Impact of Living with Ulcerative Colitis-Patients' and Physicians' View. *Inflammatory Bowel Diseases*. 2021 2021;27(11):1747-1755. doi:doi:

24. Dubinsky MC, Irving PM, Panaccione R, et al. Incorporating patient experience into drug development for ulcerative colitis: development of the Urgency Numeric Rating Scale, a patient-reported outcome measure to assess bowel urgency in adults. *J Patient Rep Outcomes*. 2022 2022;6(1):31. doi:doi:

25. Dulai PS, Singh S, Jairath V, et al. Prevalence of endoscopic improvement and remission according to patient-reported outcomes in ulcerative colitis. *Alimentary Pharmacology & Therapeutics*. 2020 2020;51(4):435-445. doi:doi:

26. Dulai PS, Jairath V, Khanna R, et al. Development of the symptoms and impacts questionnaire for Crohn's disease and ulcerative colitis. *Aliment Pharmacol Ther*. 2020 2020;51(11):1047-1066. doi:doi:

27. Engel K, Homsi M, Suzuki R, et al. Newly Diagnosed Patients with Inflammatory Bowel Disease: The Relationship Between Perceived Psychological Support, Health-Related Quality of Life, and Disease Activity. *Health Equity*. 2021 2021;5(1):42-48. doi:doi:

28. Frigstad SO, Høivik ML, Jahnsen J, et al. Pain Severity and Vitamin D Deficiency in IBD Patients. *Nutrients*. 2019 2019;12(1)doi:doi:

29. Ghosh S, Bressler B, Petkau J, et al. Healthcare Providers Underestimate Patients’ Glucocorticoid Use in Crohn’s Disease. *Digestive Diseases and Sciences*. 2019 2019;64(5):1142-1149. doi:doi:

30. Gollifer RM, Menys A, Makanyanga J, et al. Relationship between MRI quantified small bowel motility and abdominal symptoms in Crohn's disease patients-a validation study. *Br J Radiol*. 2018 2018;91(1089):20170914. doi:doi:

31. Gong W, Guo K, Zheng T, et al. JINLING (Judicious INdex of Luminal INflammation Grade) score, an effective indicator to assess inflammation severity in Crohn's disease. *Eur J Gastroenterol Hepatol*. 2021 2021;33(8):1049-1054. doi:doi:

32. Gorrepati VS, Soriano C, Johri A, et al. Abdominal Pain and Anxious or Depressed State Are Independently Associated With Weight Loss in Inflammatory Bowel Disease. *Crohns & Colitis 360*. 2020 2020;2(2)doi:doi:

33. Gracie DJ, Hamlin PJ, Ford AC. Longitudinal impact of IBS-type symptoms on disease activity, healthcare utilization, psychological health, and quality of life in inflammatory bowel disease. *American Journal of Gastroenterology*. 2018 2018;113(5):702-712. doi:doi:

34. Grant A, Lerer T, Griffiths AM, Hyams JS, Otley A. Assessing disease activity using the pediatric Crohn's disease activity index: Can we use subjective or objective parameters alone? *World J Gastroenterol*. 2021;27(30):5100-5111.

35. Halloran J, McDermott B, Ewais T, et al. Psychosocial burden of inflammatory bowel disease in adolescents and young adults. *Intern Med J*. 2021 2021;51(12):2027-2033. doi:doi:

36. Hisamatsu T, Suzuki Y, Kobayashi M, et al. Long-term safety and effectiveness of adalimumab in Japanese patients with Crohn's disease: 3-year results from a real-world study. *Intestinal Research*. 2021 2021;19(4):408-418. doi:doi:

37. Hoekman DR, Vlieger AM, Stokkers PC, et al. Hypnotherapy for Irritable Bowel Syndrome-Type Symptoms in Patients with Quiescent Inflammatory Bowel Disease: A Randomized, Controlled Trial. *Journal of Crohns & Colitis*. 2021 2021;15(7):1106-1113. doi:doi:

38. Hueppe A, Langbrandtner J, Haeuser W, Raspe H, Bokemeyer B. Validation of the "German Inflammatory Bowel Disease Activity Index (GIBDI)": An Instrument for Patient-Based Disease Activity Assessment in Crohn's Disease and Ulcerative Colitis. *Zeitschrift Fur Gastroenterologie*. 2018 2018;56(10):1267-1275. doi:doi:

39. Irving PM, Iqbal T, Nwokolo C, et al. A Randomized, Double-blind, Placebo-controlled, Parallel-group, Pilot Study of Cannabidiol-rich Botanical Extract in the Symptomatic Treatment of Ulcerative Colitis. *Inflammatory Bowel Diseases*. 2018 2018;24(4):714-724. doi:doi:

40. Janssen L, Romberg-Camps M, van Bodegraven A, et al. Control Crohn Safe with episodic adalimumab monotherapy as first-line treatment study (CoCroS): study protocol for a randomised controlled trial. *BMJ Open*. 2021 2021;11(5):e042885. doi:doi:

41. Jain A, Nguyen NH, Proudfoot JA, et al. Impact of Obesity on Disease Activity and Patient-Reported Outcomes Measurement Information System (PROMIS) in Inflammatory Bowel Diseases. *Am J Gastroenterol*. 2019 2019;114(4):630-639. doi:doi:

42. Jorgensen KK, Goll GL, Sexton J, et al. Efficacy and Safety of CT-P13 in Inflammatory Bowel Disease after Switching from Originator Infliximab: Exploratory Analyses from the NOR-SWITCH Main and Extension Trials. *Biodrugs*. 2020 2020;34(5):681-694. doi:doi:

43. Kaazan P, Li T, Seow W, et al. Assessing effectiveness and patient perceptions of a novel electronic medical record for the management of inflammatory bowel disease. *JGH Open*. 2021 2021;5(9):1063-1070. doi:doi:

44. Kaimakliotis P, Ramadugu A, Kang J, et al. Targeted housestaff intervention reduces opioid use without worsening patient-reported pain scores and improves outcomes among patients with IBD: the “IBD pain ladder”. *International Journal of Colorectal Disease*. 2021 2021;36(6):1193-1200. doi:doi:

45. Kamal N, Motwani K, Wellington J, Wong U, Cross RK. Fecal incontinence in inflammatory bowel disease. *Crohn's and Colitis 360*. 2021 2021;3(2)doi:doi:

46. Kani HT, Ergenc I, Arikan H, et al. Validation and reliability of the Turkish version of the inflammatory bowel disease questionnaire for ulcerative colitis and Crohn's disease. *Turkish Journal of Gastroenterology*. 2020 2020;31(8):566-572. doi:doi:

47. Keller R, Mazurak N, Fantasia L, et al. Quality of life in inflammatory bowel diseases: it is not all about the bowel. *Intest Res*. 2021 2021;19(1):45-52. doi:doi:

48. Khera AJ, Chase JW, Salzberg M, Thompson AJV, Kamm MA. Gut-Directed Pelvic Floor Behavioral Treatment for Fecal Incontinence and Constipation in Patients with Inflammatory Bowel Disease. *Inflamm Bowel Dis*. 2019 2019;25(3):620-626. doi:doi:

49. Kim AH, Roberts C, Feagan BG, et al. Developing a Standard Set of Patient-Centred Outcomes for Inflammatory Bowel Disease-an International, Cross-disciplinary Consensus. *J Crohns Colitis*. Mar 28 2018;12(4):408-418. doi:10.1093/ecco-jcc/jjx161

50. Kim B, Chae J, Kim EH, et al. Physical activity and quality of life of patients with inflammatory bowel disease. *Medicine (United States)*. 2021 2021;100(27)doi:doi:

51. Kim ES, Park KS, Cho KB, et al. Development of a Web-based, self-reporting symptom diary for Crohn's Disease, and its correlation with the Crohn's Disease Activity Index. *J Crohns Colitis*. 2017 2017;11(12):1449-1455. doi:doi:

52. Kim ES, Kim SK, Jang BI, et al. Disease Activity Patterns Recorded Using a Mobile Monitoring System Are Associated with Clinical Outcomes of Patients with Crohn's Disease. *Dig Dis Sci*. 2018 2018;63(9):2220-2230. doi:doi:

53. Koch AK, Schöls M, Langhorst J, Dobos G, Cramer H. Perceived stress mediates the effect of yoga on quality of life and disease activity in ulcerative colitis. Secondary analysis of a randomized controlled trial. *J Psychosom Res*. 2020 2020;130:109917. doi:doi:

54. Kochar B, Long MD, Galanko J, Raffals LE, Ananthakrishnan A, Sandler RS. Inflammatory Bowel Disease is Similar in Patients with Older Onset and Younger Onset. *Inflammatory Bowel Diseases*. 2017 2017;23(7):1187-1194. doi:doi:

55. Kruis W, Siegmund B, Lesniakowski K, et al. Novel Budesonide Suppository and Standard Budesonide Rectal Foam Induce High Rates of Clinical Remission and Mucosal Healing in Active Ulcerative Proctitis, a Randomised, Controlled, Non-inferiority Trial. *Journal of Crohn's & colitis*. 2022 2022;doi:doi:

56. Kumar A, Kim ES, Kozan P, et al. Patient-Preferences Favoring Treatment Discontinuation Are Reduced with Vedolizumab and Ustekinumab Compared with TNF Antagonists in Inflammatory Bowel Disease. *Crohn's and Colitis 360*. 2020 2020;2(4):1-9. doi:doi:

57. Lahat A, Falach-Malik A, Haj O, Shatz Z, Ben-Horin S. Change in bowel habits during menstruation: are IBD patients different? *Therapeutic Advances in Gastroenterology*. 2020 2020;13doi:doi:

58. Larussa T, Flauti D, Abenavoli L, et al. The Reality of Patient-Reported Outcomes of Health-Related Quality of Life in an Italian Cohort of Patients with Inflammatory Bowel Disease: Results from a Cross-Sectional Study. *J Clin Med*. 2020 2020;9(8)doi:doi:

59. Le Berre C, Bourreille A, Flamant M, et al. Protocol of a multicentric prospective cohort study for the VALIDation of the IBD-disk instrument for assessing disability in inflammatory bowel diseases: the VALIDate study. *BMC Gastroenterol*. 2020 2020;20(1):110. doi:doi:

60. Lee A, Moulton D, McKernan L, et al. Clinical Hypnosis in Pediatric Crohn's Disease: A Randomized Controlled Pilot Study. *J Pediatr Gastroenterol Nutr*. 2021 2021;72(3):e63-e70. doi:doi:

61. Li J, Sun Y, Hu X, et al. Impact of the SARS-CoV-2 Delta Variant on the Psychological States and Health-Related Quality of Life in Patients With Crohn's Disease. *Frontiers in Medicine*. 2022 2022;9doi:doi:

62. Lightner AL, Dadgar N, Matyas C, et al. A Phase IB/IIA study of remestemcel-L, an allogeneic bone marrow derived mesenchymal stem cell product, for the treatment of medically refractory ulcerative colitis: An interim analysis. *Colorectal Dis*. 2022 2022;doi:doi:

63. Lo B, Julsgaard M, Vester-Andersen MK, Vind I, Burisch J. Disease activity, steroid use and extraintestinal manifestation are associated with increased disability in patients with inflammatory bowel disease using the inflammatory bowel disease disability index: A cross-sectional multicentre cohort study. *European Journal of Gastroenterology and Hepatology*. 2018 2018;30(10):1130-1136. doi:doi:

64. Loftus EV, Reinisch W, Panaccione R, et al. Adalimumab Effectiveness Up to Six Years in Adalimumab-naïve Patients with Crohn's Disease: Results of the PYRAMID Registry. *Inflamm Bowel Dis*. 2019 2019;25(9):1522-1531. doi:doi:

65. Ludvigsson JF, Andersson M, Bengtsson J, et al. Swedish Inflammatory Bowel Disease Register (SWIBREG) - a nationwide quality register. *Scand J Gastroenterol*. 2019 2019;54(9):1089-1101. doi:doi:

66. Marcovitch L, Nissan A, Mack D, et al. Item Generation and Reduction Toward Developing a Patient-reported Outcome for Pediatric Ulcerative Colitis (TUMMY-UC). *J Pediatr Gastroenterol Nutr*. 2017 2017;64(3):373-377. doi:doi:

67. Marcovitch L, Focht G, Horesh A, et al. Agreement on Symptoms Between Children With Ulcerative Colitis and Their Caregivers: Towards Developing the TUMMY-UC. *J Pediatr Gastroenterol Nutr*. 2021 2021;73(2):e35-e38. doi:doi:

68. Marinelli C, Savarino E, Inferrera M, et al. Factors Influencing Disability and Quality of Life during Treatment: A Cross-Sectional Study on IBD Patients. *Gastroenterology Research and Practice*. 2019 2019;2019doi:doi:

69. Marrie RA, Graff LA, Fisk JD, Patten SB, Bernstein CN. The Relationship Between Symptoms of Depression and Anxiety and Disease Activity in IBD Over Time. *Inflamm Bowel Dis*. 2021 2021;27(8):1285-1293. doi:doi:

70. Masoodi M, Mahdiabadi MA, Mokhtare M, et al. The efficacy of curcuminoids in improvement of ulcerative colitis symptoms and patients' self-reported well-being: A randomized double-blind controlled trial. *J Cell Biochem*. 2018 2018;119(11):9552-9559. doi:doi:

71. Melmed GY, Oliver B, Hou JK, et al. Quality of Care Program Reduces Unplanned Health Care Utilization in Patients With Inflammatory Bowel Disease. *American Journal of Gastroenterology*. 2021 2021;116(12):2410-2418. doi:doi:

72. Molander P, Ylänne K. Impact of ulcerative colitis on patients’ lives: results of the Finnish extension of a global ulcerative colitis narrative survey. *Scandinavian Journal of Gastroenterology*. 2019 2019;54(7):869-875. doi:doi:

73. Moon JR, Lee CK, Hong SN, et al. Unmet Psychosocial Needs of Patients with Newly Diagnosed Ulcerative Colitis: Results from the Nationwide Prospective Cohort Study in Korea. *Gut Liver*. 2020 2020;14(4):459-467. doi:doi:

74. Moum KM, Moum B, Opheim R. Patients with inflammatory bowel disease on immunosuppressive drugs: perspectives' on COVID-19 and health care service during the pandemic. *Scand J Gastroenterol*. 2021 2021;56(5):545-551. doi:doi:

75. Murphy LK, Rights JD, Ricciuto A, Church PC, Ahola Kohut S. Biopsychosocial Correlates of Presence and Intensity of Pain in Adolescents With Inflammatory Bowel Disease. *Front Pediatr*. 2020 2020;8:559. doi:doi:

76. Naegeli AN, Hunter T, Dong Y, et al. Full, Partial, and Modified Permutations of the Mayo Score: Characterizing Clinical and Patient-Reported Outcomes in Ulcerative Colitis Patients. *Crohn's and Colitis 360*. 2021 2021;3(1)doi:doi:

77. Nguyen NH, Zhang X, Long MD, Sandborn WJ, Kappelman MD, Singh S. Patient-Reported Outcomes and Risk of Hospitalization and Readmission in Patients with Inflammatory Bowel Diseases. *Dig Dis Sci*. 2022 2022;67(6):2039-2048. doi:doi:

78. Nishida Y, Hosomi S, Fujimoto K, et al. Impact of the Lockdown Due to the COVID-19 Pandemic on Patients With Inflammatory Bowel Disease. *Front Med (Lausanne)*. 2021 2021;8:649759. doi:doi:

79. Norton C, Syred J, Kerry S, et al. Supported online self-management versus care as usual for symptoms of fatigue, pain and urgency/incontinence in adults with inflammatory bowel disease (IBD-BOOST): study protocol for a randomised controlled trial. *Trials*. 2021 2021;22(1):516. doi:doi:

80. Olden KW, Chey WD, Shringarpure R, Paul Nicandro J, Chuang E, Earnest DL. Alosetron versus traditional pharmacotherapy in clinical practice: effects on resource use, health-related quality of life, safety and symptom improvement in women with severe diarrhea-predominant irritable bowel syndrome. *Current Medical Research and Opinion*. 2019 2019;35(3):461-472. doi:doi:

81. Ostromohov G, Fibelman M, Hirsch A, et al. Assessment of patients' understanding of inflammatory bowel diseases: Development and validation of a questionnaire. *United European Gastroenterol J*. 2022 2022;10(1):104-114. doi:doi:

82. Omori T, Saruta M, Nagaki A, et al. Real-world safety and efficacy of twice-daily budesonide 2-mg foam in patients with ulcerative colitis: interim analysis of post-marketing surveillance. *Expert Opin Pharmacother*. 2021 2021;22(11):1505-1511. doi:doi:

83. Opheim R, Moum B, Grimstad BT, et al. Self-esteem in patients with inflammatory bowel disease. *Quality of Life Research*. 2020 2020;29(7):1839-1846. doi:doi:

84. Otto-Sobotka F, Peplies J, Timmer A. Modeling determinants of satisfaction with health care in youth with inflammatory bowel disease part 2: semiparametric distributional regression. *Clin Epidemiol*. 2019 2019;11:403-417. doi:doi:

85. Panés J, Vermeire S, Lindsay JO, et al. Tofacitinib in Patients with Ulcerative Colitis: Health-Related Quality of Life in Phase 3 Randomised Controlled Induction and Maintenance Studies. *J Crohns Colitis*. 2018 2018;12(2):145-156. doi:doi:

86. Panés J, D’Haens GR, Higgins PDR, et al. Long-term safety and tolerability of oral tofacitinib in patients with Crohn’s disease: results from a phase 2, open-label, 48-week extension study. *Alimentary Pharmacology and Therapeutics*. 2019 2019;49(3):265-276. doi:doi:

87. Paramsothy S, Kamm MA, Kaakoush NO, et al. Multidonor intensive faecal microbiota transplantation for active ulcerative colitis: a randomised placebo-controlled trial. *Lancet*. 2017 2017;389(10075):1218-1228. doi:doi:

88. Parra Izquierdo V, Frias-Ordonez J, Galindo P, Romero-Sanchez C, Florez C. P056Real life experience with the use of tofacitinib in ulcerative colitis in Colombia: case series. *The American journal of gastroenterology*. 2021 2021;116:S14-S15. doi:doi:

89. Parra RS, Chebli JMF, Amarante H, et al. Quality of life, work productivity impairment and healthcare resources in inflammatory bowel diseases in Brazil. *World J Gastroenterol*. 2019 2019;25(38):5862-5882. doi:doi:

90. Pittet V, Vaucher C, Froehlich F, Burnand B, Michetti P, Maillard MH. Patient self-reported concerns in inflammatory bowel diseases: A gender-specific subjective quality-of-life indicator. *PLoS One*. 2017 2017;12(2):e0171864. doi:doi:

91. Pittet V, Vaucher C, Froehlich F, Maillard MH, Michetti P. Patient-reported healthcare expectations in inflammatory bowel diseases. *PLoS One*. 2018 2018;13(5):e0197351. doi:doi:

92. Pittet VEH, Maillard MH, Simonson T, Fournier N, Rogler G, Michetti P. Differences in Outcomes Reported by Patients With Inflammatory Bowel Diseases vs Their Health Care Professionals. *Clin Gastroenterol Hepatol*. 2019 2019;17(10):2050-2059.e1. doi:doi:

93. Probert CS, Sebastian S, Gaya DR, et al. Golimumab induction and maintenance for moderate to severe ulcerative colitis: results from GO-COLITIS (Golimumab: a Phase 4, UK, open label, single arm study on its utilization and impact in ulcerative Colitis). *BMJ Open Gastroenterol*. 2018 2018;5(1):e000212. doi:doi:

94. Raffals LE, Saha S, Bewtra M, et al. The Development and Initial Findings of A Study of a Prospective Adult Research Cohort with Inflammatory Bowel Disease (SPARC IBD). *Inflammatory Bowel Diseases*. 2022 2022;28(2):192-199. doi:doi:

95. Ramos A, Vergara M, Melcarne L, Sicilia B, Gomollón F, Calvet X. Validation of a self-reported work disability questionnaire for ulcerative colitis. *Medicine (United States)*. 2018 2018;97(39)doi:doi:

96. Rassart J, Van Wanseele C, Debrun L, et al. Illness Identity in Inflammatory Bowel Disease. *International Journal of Behavioral Medicine*. 2022 2022;doi:doi:

97. Regev S, Odes S, Slonim-Nevo V, et al. Normative data for the Brief Symptom Inventory for patients with Crohn's disease. *Psychology & Health*. 2022 2022;37(2):246-257. doi:doi:

98. Ricci L, Epstein J, Buisson A, et al. Flare-IBD: development and validation of a questionnaire based on patients' messages on an internet forum for early detection of flare in inflammatory bowel disease: study protocol. *BMJ Open*. 2020 2020;10(7):e037211. doi:doi:

99. Sahnan K, Tozer PJ, Adegbola SO, et al. Developing a core outcome set for fistulising perianal Crohn's disease. *Gut*. 2019 2019;68(2):226-238. doi:doi:

100. Sands BE, Han C, Gasink C, et al. The Effects of Ustekinumab on Health-related Quality of Life in Patients With Moderate to Severe Crohn's Disease. *J Crohns Colitis*. 2018 2018;12(8):883-895. doi:doi:

101. Sattoe JNT, Peeters MAC, Haitsma J, van Staa A, Wolters VM, Escher JC. Value of an outpatient transition clinic for young people with inflammatory bowel disease: a mixed-methods evaluation. *BMJ Open*. 2020 2020;10(1):e033535. doi:doi:

102. Schäfer SK, Weidner KJ, Hoppner J, et al. Design and validation of a German version of the GSRS-IBS - an analysis of its psychometric quality and factorial structure. *BMC Gastroenterol*. 2017 2017;17(1):139. doi:doi:

103. Sekhri S, Rao B, Mohananey A, et al. Serum trough levels of infliximab are not associated with peripheral arthralgia activity in patients with inflammatory bowel disease. *BMJ Open Gastroenterol*. 2021 2021;8(1)doi:doi:

104. Shah R, Wright E, Tambakis G, et al. Telehealth model of care for outpatient inflammatory bowel disease care in the setting of the COVID-19 pandemic. *Internal Medicine Journal*. 2021 2021;51(7):1038-1042. doi:doi:

105. Shaw AL, Tomanelli A, Bradshaw TP, Petschow BW, Burnett BP. Impact of serum-derived bovine immunoglobulin/protein isolate therapy on irritable bowel syndrome and inflammatory bowel disease: a survey of patient perspective. *Patient Prefer Adherence*. 2017 2017;11:1001-1007. doi:doi:

106. Shih S, Cushman G, Reed B. Temperament and Health-Related Quality of Life in Newly Diagnosed Pediatric Inflammatory Bowel Disease. *Journal of pediatric psychology*. 2021 2021;46(4):404-412. doi:doi:

107. Smyth M, Chan J, Evans K, et al. Cross-Sectional Analysis of Quality of Life in Pediatric Patients with Inflammatory Bowel Disease in British Columbia, Canada. *J Pediatr*. 2021 2021;238:57-65.e2. doi:doi:

108. Spagnuolo R, Larussa T, Iannelli C, et al. COVID-19 and inflammatory bowel disease: Patient knowledge and perceptions in a single center survey. *Medicina (Lithuania)*. 2020 2020;56(8):1-11. doi:doi:

109. Stawowczyk E, Kawalec P, Kowalska-Duplaga K, Mossakowska M. Productivity Loss Among Parents of Children With Inflammatory Bowel Diseases in Relation to Disease Activity and Patient's Quality of Life. *J Pediatr Gastroenterol Nutr*. 2020 2020;71(3):340-345. doi:doi:

110. Svolos V, Gerasimidis K, Buchanan E, et al. Dietary treatment of Crohn's disease: perceptions of families with children treated by exclusive enteral nutrition, a questionnaire survey. *BMC Gastroenterol*. 2017 2017;17(1):14. doi:doi:

111. Taft TH, Quinton S, Jedel S, Simons M, Mutlu EA, Hanauer SB. Posttraumatic Stress in Patients With Inflammatory Bowel Disease: Prevalence and Relationships to Patient-Reported Outcomes. *Inflamm Bowel Dis*. 2022 2022;28(5):710-719. doi:doi:

112. Taxonera C, Martínez-Montiel MP, Barreiro-de-Acosta M, et al. Preferences and satisfaction of IBD patients after switching from adalimumab 40 mg weekly to 80 mg every other week given as a single injection: the ADASCAL study. *Therap Adv Gastroenterol*. 2021 2021;14:17562848211056157. doi:doi:

113. Teich N, Schulze H, Knop J, Obermeier M, Stallmach A. Novel Approaches Identifying Relevant Patient-Reported Outcomes in Patients With Inflammatory Bowel Diseases-LISTEN. *Crohns & Colitis 360*. 2021 2021;3(3)doi:doi:

114. Teich N, Bläker M, Holtkamp-Endemann F, Jörgensen E, Stallmach A, Hohenberger S. Effect of Originator Infliximab Treatment on Disease-Related Hospitalizations, Work Productivity and Activity Impairment, and Health Resource Utilization in Patients with Crohn's Disease in a Real-Life Setting: Results of a Prospective Multicenter Study in Germany. *Inflamm Intest Dis*. 2021 2021;6(1):48-60. doi:doi:

115. Thomas PWA, den Broeder N, Derikx M, et al. Impact of Biological Therapies and Tofacitinib on Real-world Work Impairment in Inflammatory Bowel Disease Patients: A Prospective Study. *Inflamm Bowel Dis*. 2022 2022;doi:doi:

116. Tiankanon K, Limsrivilai J, Poocharoenwanich N, et al. Burden of Inflammatory Bowel Disease on Patient Mood, Fatigue, Work, and Health-Related Quality of Life in Thailand: A Case-Control Study. *Crohns & Colitis 360*. 2021 2021;3(4)doi:doi:

117. Timmer A, Stark R, Peplies J, Classen M, Laass MW, Koletzko S. Current health status and medical therapy of patients with pediatric-onset inflammatory bowel disease: a survey-based analysis on 1280 patients aged 10-25 years focusing on differences by age of onset. *European Journal of Gastroenterology & Hepatology*. 2017 2017;29(11):1276-1283. doi:doi:

118. Tormey LK, Reich J, Chen YS, et al. Limited Health Literacy Is Associated With Worse Patient-Reported Outcomes in Inflammatory Bowel Disease. *Inflamm Bowel Dis*. 2019 2019;25(1):204-212. doi:doi:

119. Trindade IA, Ferreira C, Moura-Ramos M, Pinto-Gouveia J. An 18-month study of the effects of IBD symptomatology and emotion regulation on depressed mood. *International Journal of Colorectal Disease*. 2017 2017;32(5):651-660. doi:doi:

120. Trivedi I, Darguzas E, Balbale SN, et al. Patient Understanding of "Flare" and "Remission" of Inflammatory Bowel Disease. *Gastroenterol Nurs*. 2019 2019;42(4):375-385. doi:doi:

121. Tsuda S, Kunisaki R, Kato J, et al. Patient self-reported symptoms using visual analog scales are useful to estimate endoscopic activity in ulcerative colitis. *Intest Res*. 2018 2018;16(4):579-587. doi:doi:

122. Vollebregt PF, Visscher AP, van Bodegraven AA, Felt-Bersma RJF. Validation of Risk Factors for Fecal Incontinence in Patients With Crohn's Disease. *Diseases of the Colon & Rectum*. 2017 2017;60(8):845-851. doi:doi:

123. Watanabe C, Nagahori M, Fujii T, et al. Non-adherence to Medications in Pregnant Ulcerative Colitis Patients Contributes to Disease Flares and Adverse Pregnancy Outcomes. *Digestive Diseases and Sciences*. 2021 2021;66(2):577-586. doi:doi:

124. Wei S-C, Tung C-C, Weng M-T, Wong J-M. Experience of patients with inflammatory bowel disease in using a home fecal calprotectin test as an objective reported outcome for self-monitoring. *Intestinal Research*. 2018 2018;16(4):546-553. doi:doi:

125. Weizman AV, Bressler B, Seow CH, et al. Providing Hospitalized Ulcerative Colitis Patients With Practice Guidelines Improves Patient-Reported Outcomes. *J Can Assoc Gastroenterol*. 2021 2021;4(3):131-136. doi:doi:

126. Wiestler M, Kockelmann F, Kück M, et al. Quality of Life Is Associated With Wearable-Based Physical Activity in Patients With Inflammatory Bowel Disease: A Prospective, Observational Study. *Clin Transl Gastroenterol*. 2019 2019;10(11):e00094. doi:doi:

127. Williams A-J, Paramsothy R, Wu N, et al. Australia IBD Microbiome (AIM) Study: protocol for a multicentre longitudinal prospective cohort study. *Bmj Open*. 2021 2021;11(2)doi:doi:

128. Willian MK, D'Haens G, Yarlas A, Joshi AV. Changes in health-related quality of life and work-related outcomes for patients with mild-to-moderate ulcerative colitis receiving short-term and long-term treatment with multimatrix mesalamine: a prospective, open-label study. *J Patient Rep Outcomes*. 2018 2018;2:22. doi:doi:

129. Wirth MD, Robinson C, Murphy EA, Shivappa N, Hébert JR. The dietary inflammatory index is associated with gastrointestinal infection symptoms in the national health and nutrition examination survey. *Int J Food Sci Nutr*. 2020 2020;71(1):106-115. doi:doi:

130. Xu J, Chen X, Ma K, et al. Correlation Between Sleep, Life, Mood, and Diet and Severity of Inflammatory Bowel Disease in China: A Retrospective Study. *Med Sci Monit*. 2021 2021;27:e930511. doi:doi:

131. Yamabe K, Liebert R, Flores N, Pashos CL. Health-related quality of life outcomes and economic burden of inflammatory bowel disease in Japan. *Clinicoecon Outcomes Res*. 2019 2019;11:221-232. doi:doi:

132. Yan X, Qiao Y, Tong J, et al. Assessment of patient-centered outcomes (PROs) in inflammatory bowel disease (IBD): a multicenter survey preceding a cross-disciplinary (functional) consensus. *Health Qual Life Outcomes*. 2020 2020;18(1):241. doi:doi:

133. Yu M, Ye Z, Chen Y, et al. Questionnaire assessment helps the self-management of patients with inflammatory bowel disease during the outbreak of Coronavirus Disease 2019. *Aging (Albany NY)*. 2020 2020;12(13):12468-12478. doi:doi:

134. Zhang JJ, Lou DN, Ma H, Yu CH, Chen LH, Li YM. Development of a validated Chinese version of the inflammatory bowel disease disability index. *Journal of Digestive Diseases*. 2020 2020;21(1):52-58. doi:doi:

135. Zickgraf HF, Loftus P, Gibbons B, Cohen LC, Hunt MG. "If I could survive without eating, it would be a huge relief": Development and initial validation of the Fear of Food Questionnaire. *Appetite*. 2022 2022;169:105808. doi:doi:

Supplementary Table 4. Design of the included studies

| **Article** | **Main objective** | **Study design** | **Disease** | **Treatment** | **Patients (n)** | **Disease activity at baseline** | **Validation** |
| --- | --- | --- | --- | --- | --- | --- | --- |
| Bennebroek Evertsz *et al.* 2013a^1^ | To develop a patient-based HBI of CD activity and to compare it with the clinician-based HBI of CD activity in CD outpatients | Observational cross-sectional | CD | NA | CD: 181 | Not reported as such | Validated in the study |
| Bennebroek Evertsz *et al.* 2013b^2^ | To develop a patient-based SCCAI of UC activity and to compare it with the clinician-based SCCAI, CRP and PGA of UC activity. | Observational cross-sectional | UC | NA | UC: 149 | Not reported as such | Validated in the study |
| Bodger *et al.* 2013^3^ | To develop a simple, generic tool for capturing disease control from the patient’s perspective to address these barriers | Observational cohort study (prospective) | IBD | NA | CD: 160, UC: 139 | HBI: 5 (SD 5); SCCAI: 4 (3) | Validated in the study |
| Clara *et al.* 2009^4^ | To propose a single-item indicator of disease activity over an extended time, the Manitoba IBD Index (MIBDI) | Observational cohort study (prospective) | IBD | NA | CD 184; UC: 169 | Not collected | Validated in the study |
| Colombel *et al.* 2017^5^ | To establish the association between patient-reported outcomes, specifically symptom measures, and the presence of inflammation as measured by endoscopy and histology in UC | Retrospective, *post hoc* analysis | UC | NA | UC: 103 | Not reported | Validated in Jairath V. et al. 2015 Development of interim patient-reported outcome measures for the assessment of ulcerative colitis disease activity in clinical trials. Aliment Pharmacol Ther. 2015;42(10):1200–10^8^ |
| de Jong *et al.* 2019^6^ | To develop and validate a PROM to predict endoscopic disease activity | Observational cohort study (prospective) | IBD | NA | CD: 98, UC: 80 | Active disease in:   - Development phase: CD: 54 (55.1%), UC: 27 (33.8%) - Validation phase: CD: 62 (44.9%), UC: 44 (33.6%)   Calprotectin sub-study: CD: 30 (52.6%), UC: 17 (32.7%) | Validated in the study |
| Dragasevic *et al.* 2020^7^ | To investigate correlation between patients' reported symptoms and endoscopic and histological disease activity | Observational cross-sectional study | IBD | NA | CD: 63, UC: 96 | NA | Validated in Jairath V. et al. 2015 Development of interim patient-reported outcome measures for the assessment of ulcerative colitis disease activity in clinical trials. Aliment Pharmacol Ther. 2015;42(10):1200–10^8^ and  Khanna R., et al. A retrospective analysis: the development of patient reported outcome measures for the assessment of Crohn’s disease activity. Alimentary Pharmacology & Therapeutics. 2015;41(1):77–86.9^21^ |
| Golovics *et al.* 2021^9^ | To determine the correlation between PRO, clinical scores and symptoms with endoscopy and biomarkers in patients with UC | Observational cohort study (prospective) | UC | NA | 171 | ND | Validated in Jairath V. et al. 2015 Development of interim patient-reported outcome measures for the assessment of ulcerative colitis disease activity in clinical trials. Aliment Pharmacol Ther. 2015;42(10):1200–10^8^ |
| Kamat *et al.* 2022^10^ | To assess the correlation between FCP and PRO (IBD control-8 and IBD control-VAS) | Observational cross-sectional study | UC | NA | 57 | Remission: n=32, active disease: n=25 | Validated in Bodger K, et al. Development and validation of a rapid, generic measure of disease control from the patient’s perspective: the IBD-control questionnaire. Gut. 2014;63:1092–1102. ^3^ |
| Lewis *et al.*, 2020^11^ | To assess the correlation between SES-CD and individual CDAI items | *Post hoc* analysis | CD | Adalimumab, upadacitinib and risankizumab | 353 | CDAI: 306.1 (mean) | Validated in Khanna et al. A retrospective analysis: the development of patient reported outcome measures for the assessment of Crohn’s disease activity. Alimentary Pharmacology & Therapeutics. 2015;41(1):77–86.^21^ |
| Ma *et al.* 2020^12^ | To evaluate the correlation of RB and SF with endoscopic appearance in patients with mild-to-moderate UC | *Post hoc* analysis | UC | Mesalazine | 817 | MCS: 7.7 (1.3); | Validated in Jairath V. et al. 2015 Development of interim patient-reported outcome measures for the assessment of ulcerative colitis disease activity in clinical trials. Aliment Pharmacol Ther. 2015;42(10):1200–10^8^ |
| Morris *et al.* 2018^13^ | To assess the predictive ability of individual components of the CDAI, along with biomarker concentrations, to create models for predicting endoscopic disease activity | *Post hoc* analysis | CD | NA | 164 | No data (CDAI >150: active; CDAI <150: remission) | Validated in Khanna R. et al. A retrospective analysis: the development of patient reported outcome measures for the assessment of Crohn’s disease activity. Alimentary Pharmacology & Therapeutics. 2015;41(1):77–86.^21^ |
| Sexton *et al.* 2019^14^ | To develop a patient-reported outcome measure to assess a broader range of IBD symptoms | Observational cohort study (prospective) | IBD | NA | Study 1: CD: 142, UC: 125 Study 2: CD: 154 | Study 1: IBD proportion active: CD: *n* = 26 (18.7%), UC: *n*= 19 (15.6%).  Study 2: IBD proportion active: *n =*93 (60.3%) | Validated in the study |
| Subramanian *et al.* 2016^15^ | To assess the utility of a single-item, patient-reported global disease control scale by comparing it with conventional clinical indices of disease activity and evaluating its correlation with treatment escalation | Observational cross-sectional study | IBD | NA | CD: 209; UC: 196 | Patients in remission: 63% | Validated in the study |
| Surti *et al.* 2013^16^ | To assess the validity, reliability and responsiveness of an NRS and evaluate its use in clinical practice in patients with CD and UC | Observational cohort study (prospective) | IBD | NA | CD: 135; UC: 81 | Not reported | Validated in the study |
| Tow KE, *et al.* 2019^17^ | To validate a digital support tool (DST) delivered via a tablet device in an outpatient setting to assess IBD activity and psychological distress | Observational cohort study (prospective) | IBD | NA | CD: 48, UC: 33 | CD patients in remission.   - MHBI: 80%; - PRO-2-CD: 60% - PRO-3-CD: 52% - PRO-2CDSR: 48% - PRO-3CDSR: 48%   UC patients in remission.   - PMI: 72.7% - PRO-2-UC: 90.9% - PRO-2UCSR: 59.1% | Validated in Jairath V. et al. 2015 Development of interim patient-reported outcome measures for the assessment of ulcerative colitis disease activity in clinical trials. Aliment Pharmacol Ther. 2015;42(10):1200–108 and  Khanna R., et al. A retrospective analysis: the development of patient reported outcome measures for the assessment of Crohn’s disease activity. Alimentary Pharmacology & Therapeutics. 2015;41(1):77–86.^21^ |
| Van Deen *et al.* 2016^18^ | To test the ability of an IBD monitoring tool, which can be used with mobile technologies, to assess disease activity in patients with CD or UC | Observational cohort study (prospective) | IBD | NA | CD: 301; UC: 265 | Active disease:  Development:   - CD: 30% - UC: 34%   Validation:   - CD: 27% - UC: 31% | Validated in the study |
| Vicente Lidón R, *et al.* 2021^19^ | To translate IBD-Control into Spanish, and to adapt and validate it | Observational cohort study (prospective) | IBD | NA | All patients in basal visit. CD: 177, UC: 71, unclassified: 1. Follow-up. CD: 71, UC: 26 | Only in follow-up group, all patients. First visit.   - IBD-Control: 16.04 (6.33) - IBD-Control-VAS: 75.16 (22.88) - IBD-Control-8: 10.35 (4.06) | Validated in Bodger K, et al. Development and validation of a rapid, generic measure of disease control from the patient’s perspective: the IBD-control questionnaire. Gut. 2014;63:1092–1102 ^3^ |
| Zittan E, *et al.* 2017^20^ | To develop the HBI-PRO and to assess its correlation with endoscopic disease activity using SES-CD | Observational cohort study (prospective) | CD | NA | 88 (of these, 48 were not further operated) | CD cohort (non-operated):   - Clinical remission (HBI < 5): *n* = 39 (81.3%). - Mild clinical activity (HBI 5–7): *n* = 3 (6.3%). - Moderate activity (HBI 8–16): *n* = 6 (12.5%).   Post-op cohort:   - Clinical remission (HBI < 5): *n* = 30 (75.0%). - Mild clinical activity (HBI 5–7): *n* = 5 (12.5%). - Moderate activity (HBI 8–16): *n* = 5 (12.5%). - Rutgeerts score > 1: - no: *n* = 19 (47.5%) - yes: *n* = 21 (52.5%) | Validated in the study. |
| AP; abdominal pain; CD, Crohn’s disease; CDAI, Crohn’s Disease Activity Index; CRP, C-reactive protein; FCP, faecal calprotectin; HBI, Harvey-Bradshaw Index; MCS, Mayo Clinic Score; MES, Mayo endoscopic subscore; MIBDI, Manitoba IBD Index; PGA, Physician’s global assessment; pMCS, partial Mayo Clinic Score; PRO-2, two-item patient-reported outcome; ; RB, rectal bleeding; SCCAI, Simple Clinical Colitis Activity Index; SES, Simple Endoscopic Score; SF, stool frequency; UC, ulcerative colitis | | | | | | |  |

**References**

1. Bennebroek Evertsz F, Hoeks CC, Nieuwkerk PT, et al. Development of the patient Harvey Bradshaw index and a comparison with a clinician-based Harvey Bradshaw index assessment of Crohn's disease activity. *J Clin Gastroenterol*. Nov-Dec 2013;47(10):850-6. doi:10.1097/MCG.0b013e31828b2196

2. Bennebroek Evertsz F, Nieuwkerk PT, Stokkers PC, et al. The patient simple clinical colitis activity index (P-SCCAI) can detect ulcerative colitis (UC) disease activity in remission: a comparison of the P-SCCAI with clinician-based SCCAI and biological markers. *J Crohns Colitis*. Dec 2013;7(11):890-900. doi:10.1016/j.crohns.2012.11.007

3. Bodger K, Ormerod C, Shackcloth D, Harrison M, Collaborative IBDC. Development and validation of a rapid, generic measure of disease control from the patient's perspective: the IBD-control questionnaire. *Gut*. Jul 2014;63(7):1092-102. doi:10.1136/gutjnl-2013-305600

4. Clara I, Lix LM, Walker JR, et al. The Manitoba IBD Index: evidence for a new and simple indicator of IBD activity. *Am J Gastroenterol*. Jul 2009;104(7):1754-63. doi:10.1038/ajg.2009.197

5. Colombel JF, Keir ME, Scherl A, et al. Discrepancies between patient-reported outcomes, and endoscopic and histological appearance in UC. *Gut*. 2017 2017;66(12):2063-2068. doi:doi:

6. de Jong MJ, Roosen D, Degens J, et al. Development and Validation of a Patient-reported Score to Screen for Mucosal Inflammation in Inflammatory Bowel Disease. *J Crohns Colitis*. Apr 26 2019;13(5):555-563. doi:10.1093/ecco-jcc/jjy196

7. Dragasevic S, Sokic-Milutinovic A, Lalosevic MS, et al. Correlation of Patient-Reported Outcome (PRO-2) with Endoscopic and Histological Features in Ulcerative Colitis and Crohn's Disease Patients. *Gastroenterology Research and Practice*. 2020 2020;2020doi:doi:

8. Jairath V, Khanna R, Zou GY, et al. Development of interim patient-reported outcome measures for the assessment of ulcerative colitis disease activity in clinical trials. *Aliment Pharmacol Ther*. Nov 2015;42(10):1200-10. doi:10.1111/apt.13408

9. Golovics PA, Gonczi L, Reinglas J, et al. Patient-Reported Outcome and Clinical Scores Are Equally Accurate in Predicting Mucosal Healing in Ulcerative Colitis: A Prospective Study. *Dig Dis Sci*. Jul 2022;67(7):3089-3095. doi:10.1007/s10620-021-07178-w

10. Kamat N, Vuyyuru SK, Kedia S, et al. Correlation of fecal calprotectin and patient-reported outcome measures in patients with ulcerative colitis. *Intest Res*. Apr 2022;20(2):269-273. doi:10.5217/ir.2021.00064

11. Lewis JD, Rutgeerts P, Feagan BG, et al. Correlation of Stool Frequency and Abdominal Pain Measures With Simple Endoscopic Score for Crohn's Disease. *Inflamm Bowel Dis*. Jan 6 2020;26(2):304-313. doi:10.1093/ibd/izz241

12. Ma C, Sandborn WJ, D'Haens GR, et al. Discordance Between Patient-Reported Outcomes and Mucosal Inflammation in Patients With Mild to Moderate Ulcerative Colitis. *Clin Gastroenterol Hepatol*. 2020 2020;18(8):1760-1768.e1. doi:doi:

13. Morris MW, Stewart SA, Heisler C, et al. Biomarker-Based Models Outperform Patient-Reported Scores in Predicting Endoscopic Inflammatory Disease Activity. *Inflamm Bowel Dis*. Jan 18 2018;24(2):277-285. doi:10.1093/ibd/izx018

14. Sexton KA, Walker JR, Targownik LE, et al. The Inflammatory Bowel Disease Symptom Inventory: A Patient-report Scale for Research and Clinical Application. *Inflamm Bowel Dis*. Jul 17 2019;25(8):1277-1290. doi:10.1093/ibd/izz038

15. Subramanian S, Asher R, Weston W, et al. Validation of a Simple 0 to 10 Numerical Score (IBD-10) of Patient-reported Inflammatory Bowel Disease Activity for Routine Clinical Use. *Inflamm Bowel Dis*. Aug 2016;22(8):1902-7. doi:10.1097/MIB.0000000000000803

16. Surti B, Spiegel B, Ippoliti A, et al. Assessing health status in inflammatory bowel disease using a novel single-item numeric rating scale. *Dig Dis Sci*. May 2013;58(5):1313-21. doi:10.1007/s10620-012-2500-1

17. Tow KE, Rogge C, Lee T, Caputi P, Knowles SR. Validation of a Digital Support App to Assess Inflammatory Disease Activity and Mental Health Patient-Reported Outcomes (PROs): A Pilot Investigation. *Gastroenterol Res Pract*. 2019 2019;2019:7618468. doi:10.1155/2019/7618468

18. Van Deen WK, van der Meulen-de Jong AE, Parekh NK, et al. Development and Validation of an Inflammatory Bowel Diseases Monitoring Index for Use With Mobile Health Technologies. *Clin Gastroenterol Hepatol*. Dec 2016;14(12):1742-1750 e7. doi:10.1016/j.cgh.2015.10.035

19. Vicente Lidón R, García López S, Corsino Roche P, et al. Translation into Spanish and validation of a short questionnaire to measure the control of inflammatory bowel disease from the patient's perspective: IBD-Control, EII-Control. *Gastroenterologia y Hepatologia*. 2021 2021;doi:doi:

20. Zittan E, Kabakchiev B, Kelly OB, et al. Development of the Harvey-Bradshaw Index-pro (HBI-PRO) Score to Assess Endoscopic Disease Activity in Crohn's Disease. *J Crohns Colitis*. May 1 2017;11(5):543-548. doi:10.1093/ecco-jcc/jjw200

21. Khanna R, Zou G, D'Haens G, et al. A retrospective analysis: the development of patient reported outcome measures for the assessment of Crohn's disease activity. *Aliment Pharmacol Ther* 2015; 41: 77-86. 20141027. DOI: 10.1111/apt.13001.

Supplementary Table 5. Quality assessment of the included studies by the CASP checklist

| **Article** | **Quality level** | **CASP results^a^**  **Design** |
| --- | --- | --- |
| Clara *et al.* 2009^1^ | Moderate | ++++0000 |
| Colombel *et al.* 2017^2^ | Moderate | ++++0+0- |
| Dragasevic *et al.* 2020^3^ | Moderate | ++++0000 |
| Golovics, *et al.* 2021^4^ | Moderate | ++++0000 |
| Kamat, *et al.* 2022^5^ | Very Low | ++0+---- |
| Lewis *et al.* 2020^6^ | Moderate-Low | ++++00-- |
| Ma *et al.* 2020^7^ | Moderate-High | ++++0+00 |
| Morris *et al.* 2018^8^ | High | ++++0+++ |
| Zittan *et al.* 2017^9^ | Moderate-High | ++++0+00 |
| Bennebroek Evertsz *et al.* 2013a^10^ | Moderate | +++-++ |
| Bennebroek Evertsz *et al.* 2013b^11^ | Moderate | +++-++ |
| Bodger *et al*. 2013^12^ | Low | ++--++ |
| de Jong *et al.* 2019^13^ | Moderate | +++-++ |
| Sexton *et al.* 2019^14^ | Moderate | +0+0++ |
| Subramanian *et al.* 2016^15^ | Low | +0+0-+ |
| Surti *et al.* 2013^16^ | Moderate-Low | +0+-++ |
| Tow *et al.* 2019^17^ | Low | +0+0-+ |
| Van Deen *et al.* 2016^18^ | Moderate | +0+0++ |
| Vicente Lidón *et al.* 2021^19^ | Moderate | +++-++ |

^a^Quality assessment was performed using CASP checklists for each type of study^20^. Results depicted in the table correspond to questions related to design (questions 1–6 in the corresponding checklists). Each positive (yes) response in the questionnaire is depicted as (+), negative it is indicated as (-) and “can’t tell” is depicted as (0). An increasing number of (+) indicates a greater quality assessment score. Quality judgment has been assigned as an indicative measure (not given in the CASP assessment checklist)

**References**

1. Clara I, Lix LM, Walker JR, et al. The Manitoba IBD Index: evidence for a new and simple indicator of IBD activity. *Am J Gastroenterol*. Jul 2009;104(7):1754-63. doi:10.1038/ajg.2009.197

2. Colombel JF, Keir ME, Scherl A, et al. Discrepancies between patient-reported outcomes, and endoscopic and histological appearance in UC. *Gut*. 2017 2017;66(12):2063-2068. doi:doi:

3. Dragasevic S, Sokic-Milutinovic A, Lalosevic MS, et al. Correlation of Patient-Reported Outcome (PRO-2) with Endoscopic and Histological Features in Ulcerative Colitis and Crohn's Disease Patients. *Gastroenterology Research and Practice*. 2020 2020;2020doi:doi:

4. Golovics PA, Gonczi L, Reinglas J, et al. Patient-Reported Outcome and Clinical Scores Are Equally Accurate in Predicting Mucosal Healing in Ulcerative Colitis: A Prospective Study. *Dig Dis Sci*. Jul 2022;67(7):3089-3095. doi:10.1007/s10620-021-07178-w

5. Kamat N, Vuyyuru SK, Kedia S, et al. Correlation of fecal calprotectin and patient-reported outcome measures in patients with ulcerative colitis. *Intest Res*. Apr 2022;20(2):269-273. doi:10.5217/ir.2021.00064

6. Lewis JD, Rutgeerts P, Feagan BG, et al. Correlation of Stool Frequency and Abdominal Pain Measures With Simple Endoscopic Score for Crohn's Disease. *Inflamm Bowel Dis*. Jan 6 2020;26(2):304-313. doi:10.1093/ibd/izz241

7. Ma C, Sandborn WJ, D'Haens GR, et al. Discordance Between Patient-Reported Outcomes and Mucosal Inflammation in Patients With Mild to Moderate Ulcerative Colitis. *Clin Gastroenterol Hepatol*. 2020 2020;18(8):1760-1768.e1. doi:doi:

8. Morris MW, Stewart SA, Heisler C, et al. Biomarker-Based Models Outperform Patient-Reported Scores in Predicting Endoscopic Inflammatory Disease Activity. *Inflamm Bowel Dis*. Jan 18 2018;24(2):277-285. doi:10.1093/ibd/izx018

9. Zittan E, Kabakchiev B, Kelly OB, et al. Development of the Harvey-Bradshaw Index-pro (HBI-PRO) Score to Assess Endoscopic Disease Activity in Crohn's Disease. *J Crohns Colitis*. May 1 2017;11(5):543-548. doi:10.1093/ecco-jcc/jjw200

10. Bennebroek Evertsz F, Hoeks CC, Nieuwkerk PT, et al. Development of the patient Harvey Bradshaw index and a comparison with a clinician-based Harvey Bradshaw index assessment of Crohn's disease activity. *J Clin Gastroenterol*. Nov-Dec 2013;47(10):850-6. doi:10.1097/MCG.0b013e31828b2196

11. Bennebroek Evertsz F, Nieuwkerk PT, Stokkers PC, et al. The patient simple clinical colitis activity index (P-SCCAI) can detect ulcerative colitis (UC) disease activity in remission: a comparison of the P-SCCAI with clinician-based SCCAI and biological markers. *J Crohns Colitis*. Dec 2013;7(11):890-900. doi:10.1016/j.crohns.2012.11.007

12. Bodger K, Ormerod C, Shackcloth D, Harrison M, Collaborative IBDC. Development and validation of a rapid, generic measure of disease control from the patient's perspective: the IBD-control questionnaire. *Gut*. Jul 2014;63(7):1092-102. doi:10.1136/gutjnl-2013-305600

13. de Jong MJ, Roosen D, Degens J, et al. Development and Validation of a Patient-reported Score to Screen for Mucosal Inflammation in Inflammatory Bowel Disease. *J Crohns Colitis*. Apr 26 2019;13(5):555-563. doi:10.1093/ecco-jcc/jjy196

14. Sexton KA, Walker JR, Targownik LE, et al. The Inflammatory Bowel Disease Symptom Inventory: A Patient-report Scale for Research and Clinical Application. *Inflamm Bowel Dis*. Jul 17 2019;25(8):1277-1290. doi:10.1093/ibd/izz038

15. Subramanian S, Asher R, Weston W, et al. Validation of a Simple 0 to 10 Numerical Score (IBD-10) of Patient-reported Inflammatory Bowel Disease Activity for Routine Clinical Use. *Inflamm Bowel Dis*. Aug 2016;22(8):1902-7. doi:10.1097/MIB.0000000000000803

16. Surti B, Spiegel B, Ippoliti A, et al. Assessing health status in inflammatory bowel disease using a novel single-item numeric rating scale. *Dig Dis Sci*. May 2013;58(5):1313-21. doi:10.1007/s10620-012-2500-1

17. Tow KE, Rogge C, Lee T, Caputi P, Knowles SR. Validation of a Digital Support App to Assess Inflammatory Disease Activity and Mental Health Patient-Reported Outcomes (PROs): A Pilot Investigation. *Gastroenterol Res Pract*. 2019 2019;2019:7618468. doi:10.1155/2019/7618468

18. Van Deen WK, van der Meulen-de Jong AE, Parekh NK, et al. Development and Validation of an Inflammatory Bowel Diseases Monitoring Index for Use With Mobile Health Technologies. *Clin Gastroenterol Hepatol*. Dec 2016;14(12):1742-1750 e7. doi:10.1016/j.cgh.2015.10.035

19. Vicente Lidón R, García López S, Corsino Roche P, et al. Translation into Spanish and validation of a short questionnaire to measure the control of inflammatory bowel disease from the patient's perspective: IBD-Control, EII-Control. *Gastroenterologia y Hepatologia*. 2021 2021;doi:doi:

20. Programme CAS. Critical Appraisal Skills Programme Checklists <https://casp-uk.net/casp-tools-checklists/>

Supplementary Table 6. Description of the PROMs included in the selected articles (included items and scoring system)

| **Article** | **Disease** | **PROM** | **Domains/ítems** | **Scoring and cut-off** |
| --- | --- | --- | --- | --- |
| Bennebroek Evertsz *et al.* 2013^1^ | CD | Patient HBI | General well-being, abdominal pain, frequency of loose stools, extraintestinal manifestations, arthralgia, uveitis, erythema nodosum, pyoderma gangrenosum, aphthous ulcer, anal fissure, active fistula or abscess | HBI: ≥5 is active disease |
| De Jong *et al.* 2019^2^ | CD | MIAH-CD | Stool frequency (day), liquid or soft stool frequency, patient-reported disease activity, urgency of defecation, mucus loss, rectal bleeding, stool frequency (night), fatigue, fever, general well-being, perceived stress, weight loss, abdominal pain | Optimal cut-off value for the MIAH-CD was set at 3.6 |
| Khanna *et al*. 2015^3^ | CD | PRO-2/PRO-3 (derived from CDAI) | PRO-2: number of liquid or very loose stools (stool frequency), abdominal pain; PRO 3: number of liquid or very loose stools, abdominal pain, general well-being | Remission: stool frequency mean daily score ≤1.5, abdominal mean daily score ≤1 and general well-being score ≤1 |
| Zittan *et al.* 2017^4^ | CD | HBI-PRO | Patient’s general well-being, abdominal pain, number of liquid stools per day, abdominal mass, complications | NA |
| Bennebroek Evertsz *et al.* 2013^5^ | UC | pSCCAI | Stool frequency, nocturnal stools, urgency, adjustments in daily activities, incontinence, blood in stool, general well-being, extraintestinal manifestations, arthralgia, red or swollen joints, woken up at night from arthralgia, erythema nodosum, pyoderma gangrenosum, uveitis | SCCAI: ≥5 is active disease |
| Higgins *et al.* 2017^6^ | UC | UC-PRO/SS | Bowel signs and symptoms, abdominal symptoms, systemic symptoms, coping strategies, daily life impact, emotional impact | NA |
| Jairath *et al.* 2015^7^ | UC | PRO-2 | Rectal bleeding and number of liquid or very loose stools (stool frequency) | Remission: bleeding = 0, bowel frequency ≤2 |
| Jowet *et al.* 2003^8^ | UC | Self SCCAI | Bowel frequency during day and night, urgency, blood in stool, general well-being, extracolonic manifestations (including photographs illustrating extraintestinal manifestations) | SCCAI: >5 points is relapse |
| Bodger *et al.* 2014^9^ | IBD | IBD-Control | Disease control, symptom change, depression, fatigue, pain, night symptoms, medication-related concerns, development of new symptoms | Range of 0–16 (0 = worst control) |
| Clara *et al.* 2009^10^ | IBD | MIBDI | One single item about the frequency and persistence of IBD symptoms | 6 levels of response, dichotomous classification: active disease included experiencing symptoms constantly to occasionally (responses a to d), and inactive disease as experiencing infrequent symptoms or feeling well (responses e or f) |
| De Jong *et al.* 2019^2^ | IBD | MIAH-UC | Rectal bleeding, stool frequency (day), mucus loss, patient-reported disease activity, general well-being, liquid or soft stool frequency, urgency of defecation, weight loss, stool frequency (night), abdominal pain, perceived stress, fatigue, fever | The optimal cut-off for the MIAH-UC was set at 3.5 |
| Peyrin-Biroulet *et al.* 2012^11^ | IBD | IBD-Disk | Abdominal pain, regulating defecation. Interpersonal interactions, education and IBD work, sleep, energy, emotions, body image, sexual functions, joint pain | Range 0–100 (0 = no complaints) |
| Sexton *et al.* 2019^12^ | IBD | IBDSI long form | Bowel symptoms, abdominal discomfort, fatigue, bowel complications and systemic complications. | Score of > 24 denotes active disease for CD Score of > 17 denotes active disease for UC |
| Subramanian *et al.* 2016^13^ | IBD | IBD-10 numerical scale | A patient-reported 0 to 10 score of IBD activity (IBD-10) | Patients were asked verbally to rate on a scale of 0 to 10 their disease activity on the day of clinic attendance in answer to a standard question: “On a scale of 0 to 10, 10 being the best, how would you rate your Crohn’s disease/ulcerative colitis today?” |
| Surti *et al.* 2013^14^ | IBD | IBD NRS | Two PROs for this study, an 11-point NRS and a single self-reported “remission” item | The generic PRO of health status was measured by an 11-point NRS. This was anchored at each end (0 = “As bad as being dead”, 10 = “Perfect health”). The remission item was a yes/no question: “Do you feel your disease is in remission?” |
| Van Deen *et al.* 2016^15^ | IBD | mHI-CD and mHI-UC | Stool frequency, abdominal pain, general well-being, urgency, stool consistency, rectal bleeding, fever, anorexia, nausea/vomiting, disease activity | NA |

AP, abdominal pain; aOR, adjusted odds ratio; AUC, area under the curve; CDAI, Crohn’s Disease Activity Index; CD, Crohn’s Disease; CDEIS, Crohn’s Disease Index of Severity; CI, confidence interval; CR, clinical remission; ER, endoscopic remission; FCP, faecal calprotectin; GELS, Global Endoscopic Evaluation of Severity; GS, Geboes grading system of histological activity; GRS, Guyatt’s responsiveness statistic; HBI, Harvey–Bradshaw Index; IBD, inflammatory bowel disease; IBD-10, Inflammatory Bowel Disease-10 items; IBD NRS, Inflammatory Bowel Disease Numerical Rating Scale; IBDSI, Inflammatory Bowel Disease Symptom Inventory; MCS, Mayo Clinic Score; MES, Mayo Endoscopic Subscore; MHBI, modified Harvey–Bradshaw Index; MIAH, Monitor IBD at Home; MIBD, mastocytic inflammatory bowel disease; MIBDI, Manitoba IBD Index; NPV, negative predictive value; OR, odds ratio; pMCS, partial Mayo Clinic Score; PPV, positive predictive value; PRO-2, patient-reported outcomes 2; PRO-3, PRO-2 + FCP; PTI, Powell–Tuck Index; RB, rectal bleeding; ROC, receiver operating characteristic; SCCAI, Simple Clinical Colitis Activity Index; Self-SCAAI, Self-reported Simple Clinical Colitis Activity Index; SES, Simple Endoscopic Score; SF, stool frequency; U C-PRO/SS, Ulcerative Colitis Patient-Reported Outcomes Signs and Symptoms; UC, Ulcerative Colitis; VAS, visual analogue scale; IBD-Disk, Inflammatory Bowel Disease-Disability index.

**References**

1. Bennebroek Evertsz F, Hoeks CC, Nieuwkerk PT, et al. Development of the patient Harvey Bradshaw index and a comparison with a clinician-based Harvey Bradshaw index assessment of Crohn's disease activity. *J Clin Gastroenterol*. Nov-Dec 2013;47(10):850-6. doi:10.1097/MCG.0b013e31828b2196

2. de Jong MJ, Roosen D, Degens J, et al. Development and Validation of a Patient-reported Score to Screen for Mucosal Inflammation in Inflammatory Bowel Disease. *J Crohns Colitis*. Apr 26 2019;13(5):555-563. doi:10.1093/ecco-jcc/jjy196

3. Khanna R, Zou G, D'Haens G, et al. A retrospective analysis: the development of patient reported outcome measures for the assessment of Crohn's disease activity. *Aliment Pharmacol Ther*. Jan 2015;41(1):77-86. doi:10.1111/apt.13001

4. Zittan E, Kabakchiev B, Kelly OB, et al. Development of the Harvey-Bradshaw Index-pro (HBI-PRO) Score to Assess Endoscopic Disease Activity in Crohn's Disease. *J Crohns Colitis*. May 1 2017;11(5):543-548. doi:10.1093/ecco-jcc/jjw200

5. Bennebroek Evertsz F, Nieuwkerk PT, Stokkers PC, et al. The patient simple clinical colitis activity index (P-SCCAI) can detect ulcerative colitis (UC) disease activity in remission: a comparison of the P-SCCAI with clinician-based SCCAI and biological markers. *J Crohns Colitis*. Dec 2013;7(11):890-900. doi:10.1016/j.crohns.2012.11.007

6. Higgins PDR, Harding G, Revicki DA, et al. Development and validation of the Ulcerative Colitis patient-reported outcomes signs and symptoms (UC-pro/SS) diary. *J Patient Rep Outcomes*. 2017 2017;2(1):26. doi:10.1186/s41687-018-0049-2

7. Jairath V, Khanna R, Zou GY, et al. Development of interim patient-reported outcome measures for the assessment of ulcerative colitis disease activity in clinical trials. *Aliment Pharmacol Ther*. Nov 2015;42(10):1200-10. doi:10.1111/apt.13408

8. Jowett SL, Seal CJ, Phillips E, Gregory W, Barton JR, Welfare MR. Defining relapse of ulcerative colitis using a symptom-based activity index. *Scand J Gastroenterol*. Feb 2003;38(2):164-71. doi:10.1080/00365520310000654

9. Bodger K, Ormerod C, Shackcloth D, Harrison M, Collaborative IBDC. Development and validation of a rapid, generic measure of disease control from the patient's perspective: the IBD-control questionnaire. *Gut*. Jul 2014;63(7):1092-102. doi:10.1136/gutjnl-2013-305600

10. Clara I, Lix LM, Walker JR, et al. The Manitoba IBD Index: evidence for a new and simple indicator of IBD activity. *Am J Gastroenterol*. Jul 2009;104(7):1754-63. doi:10.1038/ajg.2009.197

11. Peyrin-Biroulet L, Hart A, Bossuyt P, et al. Etrolizumab as induction and maintenance therapy for ulcerative colitis in patients previously treated with tumour necrosis factor inhibitors (HICKORY): a phase 3, randomised, controlled trial. *Lancet Gastroenterology & Hepatology*. 2022 2022;7(2):128-140. doi:doi:

12. Sexton KA, Walker JR, Targownik LE, et al. The Inflammatory Bowel Disease Symptom Inventory: A Patient-report Scale for Research and Clinical Application. *Inflamm Bowel Dis*. Jul 17 2019;25(8):1277-1290. doi:10.1093/ibd/izz038

13. Subramanian S, Asher R, Weston W, et al. Validation of a Simple 0 to 10 Numerical Score (IBD-10) of Patient-reported Inflammatory Bowel Disease Activity for Routine Clinical Use. *Inflamm Bowel Dis*. Aug 2016;22(8):1902-7. doi:10.1097/MIB.0000000000000803

14. Surti B, Spiegel B, Ippoliti A, et al. Assessing health status in inflammatory bowel disease using a novel single-item numeric rating scale. *Dig Dis Sci*. May 2013;58(5):1313-21. doi:10.1007/s10620-012-2500-1

15. Van Deen WK, van der Meulen-de Jong AE, Parekh NK, et al. Development and Validation of an Inflammatory Bowel Diseases Monitoring Index for Use With Mobile Health Technologies. *Clin Gastroenterol Hepatol*. Dec 2016;14(12):1742-1750 e7. doi:10.1016/j.cgh.2015.10.035
